# Aberrant dopaminergic activity during consolidation causes age-related memory generalization in *Drosophila*

**DOI:** 10.1371/journal.pbio.3003752

**Published:** 2026-04-01

**Authors:** Motomi Matsuno, Nozomi Uemura, Tomoyuki Miyashita, Kyogo S. Kobayashi, Agustin Liotta, Mayumi Kimura, Kyoko Ofusa, David Owald, Naoki Matsuo, Minoru Saitoe, Junjiro Horiuchi

**Affiliations:** 1 Tokyo Metropolitan Institute of Medical Science, Higher Brain Function Project, Setagaya, Tokyo, Japan; 2 Department of Computational Biology and Medical Sciences, Graduate School of Frontier Sciences, The University of Tokyo, Kashiwa, Japan; 3 National Institute of Infectious Diseases, Department of Medical Entomology, Tokyo, Japan; 4 Department of Biology, Faculty of Science, Kyushu University, Fukuoka, Japan; 5 Institute of Neurophysiology, Charité – Universitatsmedizin Berlin, corporate member of Freie Universitat Berlin and Humboldt-Universitat zu Berlin, Berlin, Germany; 6 Department of Anesthesiology and Intensive Care, Charité – Universitatsmedizin Berlin, corporate member of Freie Universitat Berlin and Humboldt-Universitat zu Berlin, Berlin, Germany; Stony Brook University Medical Center: Stony Brook University Hospital, UNITED STATES OF AMERICA

## Abstract

Overactivation of memory networks and pathways can induce post-traumatic stress disorders and memory generalization, where memories are recalled in inappropriate situations. Here, we demonstrate that age-related defects in long-term memories in *Drosophila* are also caused by memory generalization. Aversive memory engram cells are formed in both young and old flies trained in an odor avoidance task. However, while engrams in young flies are activated specifically by odors previously paired with electrical shocks, engrams in old flies are activated by shock-paired, unpaired, and novel odors. This enhancement of engram cell activation occurs because of increased activity of dopaminergic neurons during memory consolidation in old flies. Increased dopamine signaling results from an inability of old flies to inhibit glutamatergic activation and leads to increased activation of dopamine D2 receptors on engram cells. Our data suggest that increased dopaminergic activity after training generalizes the responsiveness of engram cells to disrupt appropriate memory recall.

## Abstract 2

Long-term memories become imprecise as animals age, but the neural mechanisms of impaired memory performance during aging are not fully understood. This study shows that increased dopamine signaling during memory consolidation causes memory engram cells to respond to inappropriate cues, inducing memory generalization.

## Introduction

While animals need accurate memories, they also need to be able to generalize memories in order to respond properly to novel experiences that are similar but not identical to prior experiences [1]. Thus, a balance between memory specificity and generalization is required, with a shift in balance in either direction resulting in memory and behavioral impairments. Memory decreases with advancing age in many animals, including *Drosophila* [2]. However, it has been unclear whether altered memory generalization is a major cause of age-related memory impairments. Increases in generalization have been implicated in diseases associated with memory dysfunction, including post-traumatic stress disorder (PTSD), in which memories of past traumas can be triggered by inappropriate cues, severe depression, and anxiety [3–6].

In flies, age-related memory impairment (AMI) affects specific types of memory, including middle-term memory (MTM) and long-term memory (LTM) [2,7–9]. LTM, which lasts for over 7 days, requires increased protein synthesis and activation of transcription factors, including CREB and c-Fos, in specific neurons known as engram cells [10–12]. LTM engram cells in *Drosophila* can be identified as neurons that show training-dependent increases in Kayak, the fly homolog of c-Fos [12]. Engram cells are formed by multiple associative training trials with interspersed rest intervals (spaced training) and are reactivated during memory recall. Furthermore, the importance of c-Fos^+^ engram cells in flies has been demonstrated by showing that their inactivation prevents memory recall [12]. While some studies have examined engram cells in old animals [13,14], the relationship between memory generalization and engram cell reactivation has been unclear.

Besides neuronal transcription, LTM also requires increases in glial transcription [15]. Spaced training activates the glial transcription factor, Repo, which increases the expression of the glutamate transporter, *Eaat1* [16]. Increases in EAAT1 reduce synaptic glutamate by importing glutamate into glia. *Eaat1* expression increases shortly after spaced training, indicating that glutamate activity is normally inhibited during memory consolidation. The homophilic cell adhesion molecule, Klingon (Klg), is expressed in both neurons and glia and is necessary for spaced training-dependent activation of Repo, indicating that Klg plays an important role in transmitting neuronal activity signals to glia [17]. Interestingly, aging causes decreased expression of *Klg*, *Repo*, and *Eaat1*, suggesting that defects in glial transcription and repression of glutamate signaling may be a cause of age-related defects in LTM. Supporting this idea, increased expression of *Klg*, *Repo*, or *Eaat1* rescues age-related LTM defects [16].

Although the formation and activity of neuronal engram cells is critical for LTM, the relationship between engram cells, glial transcription, aging, and memory generalization has been unclear. Here, we determine that altered glial regulation of glutamate signaling at old ages increases activity of dopaminergic neurons that regulate engram cell connectivity. This results in memory generalization. We further find that inhibiting dopamine D2 receptor activity prevents memory generalization.

## Results

### Memory engrams are formed in aged flies, but are reactivated by inappropriate stimuli

**Fig 1:**
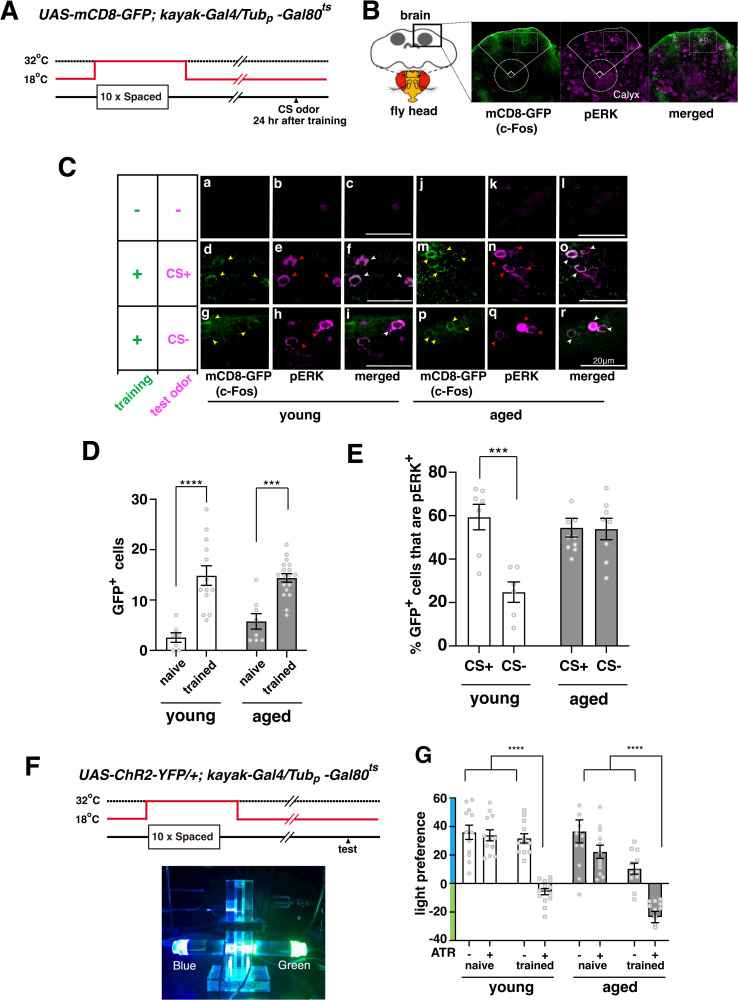
Functional memory engrams are formed in aged flies but are activated by inappropriate odors. **A)** *mCD8-GFP* was expressed under control of the *Drosophila c-Fos* (*kayak*) promoter to visualize putative engram cells. GFP signals from background *kayak* expression were inhibited using *GAL80*^*ts*^ and raising flies at permissive temperature (18 °C). 10× spaced training was performed at 32 °C to release GAL80^ts^ repression and allow training-dependent kayak to induce GFP expression. **B)** Putative engram cells were identified as GFP (cFos) signals in the indicated mushroom body cell body region dorsal to the calyx 24 hours after spaced training of *UAS-mCD8-GFP; kayak-Gal4/Tubp-Gal80*^*ts*^ flies. pERK signals observed after odor exposure 24 hours after training were used to identify cells activated during memory recall tests. **C)** Putative engram cells (mCD8-GFP signals) were observed after spaced training in young and aged flies (compare panels a and j to panels d, m, g, and p). pERK signals were observed after exposing flies to odors during memory recall tests 24 hours after training. In young flies, pERK signals after CS+ exposure overlapped significantly with GFP signals when compared to signals after CS− exposure. In aged flies, the overlap between pERK and GFP signals was similar after CS+ and CS− exposure. **D)** Quantification of putative engram cells in naïve and trained, young and aged flies. *N* = 7–18, *** indicates *P* < 0.001. **E)** Quantification of engram cell reactivation data. *N* = 6–10, *** indicates *P* < 0.001. **F)** Schematic for optogenetic reactivation of engram cells. Channelrhodopsin-YFP (ChR2-YFP) was expressed in putative engram cells using the same strategy of expressing GFP shown in (A). Twenty-four hours after spaced training, flies were tested for their preference of blue light (which activates channelrhodopsin) vs. green light. **G)** Color preferences of indicated flies. Positive color preference scores indicate that flies preferentially chose blue light over green light, while negative scores indicate that flies preferentially chose green light. *N* = 11–14, **** indicates *P* < 0.0001. Data in all bar graphs in this manuscript represent means ± SEMs. The data underlying this figure are available in S1 Data.

To determine whether age-related impairments in LTM are caused by a reduction in the formation of memory engram cells, we first examined engram cells in young (raised for 3–5 days at 18 °C) and aged flies (raised for 40 days at 18 °C) after spaced training in an odor/fear association paradigm. As previously described [12], we identified potential engram cells as GFP-positive cells located dorsally to the mushroom body calyces 24 hours after spaced training of *UAS-mCD8-GFP; kayak-Gal4/Tub*_*p*_*-Gal80*^*t*s^ flies (Fig 1A–1C). In these flies, GFP is expressed under control of a *kayak (c-Fos)* promoter. Flies were raised and aged at 18 °C to allow GAL80^ts^ to inhibit background GFP expression, and trained at the Gal80^ts^ restrictive temperature of 32 °C (Fig 1A). As seen in Fig 1C and 1D, we observed a significant increase in c-Fos-positive neurons after spaced training in both young and aged flies, suggesting that engram cells are formed to a similar extent in old flies. When we examined the reactivation of engram cells during recall using anti-pERK antibodies to detect neuronal activation upon odor exposure, we found that exposure to the shock-paired odor (CS+) reactivated a significantly larger percentage of potential engram cells compared to exposure to an unpaired odor (CS−) in young flies (Fig 1C and 1E). However, in aged flies, both the CS+ and CS− odors reactivated engram cells similarly (Fig 1C and 1E), suggesting that fear engrams are reactivated by both appropriate and inappropriate odors in aged flies. Thus, one defect of LTM in aged flies may be a recall of memories in inappropriate situations.

To verify that the c-Fos-positive cells that we identified in aged flies encode functional aversive memory engrams, we next examined whether artificial activation of these cells induces avoidance behaviors. As described previously [12], we expressed blue-light inducible channelrhodopsin in putative engram cells by training *UAS-ChR2::YFP/+; Kayak-Gal4/Tub*_*p*_*-Gal80*^*ts*^ flies at GAL80^ts^ restrictive temperature. This allowed us to activate *kayak (c-Fos)*-positive engram cells by exposing flies to blue light (Fig 1F). Young untrained flies and young flies raised in the absence of all-trans retinal (ATR), which is required for producing functional channelrhodopsin, showed a strong preference for blue light when given a choice between blue and green light (Fig 1G). However, young flies fed ATR and trained in an odor avoidance task at the GAL80^ts^ restrictive temperature lost this preference and instead showed a slight preference for green light (Fig 1G). This suggests that activation of putative aversive memory engram cells by blue light produces avoidance behaviors that are sufficient to eclipse natural light preferences. Similar to results in young flies, we found that aged trained and untrained flies raised in the absence of ATR showed a preference for blue light, while aged trained flies fed ATR showed a preference for green light. Thus, training flies in an aversive association task produces engram cells in both young and aged flies, and subsequent activation of these engram cells induces appropriate memory-associated avoidance behaviors.

### Inappropriate reactivation of engrams in aged flies is associated with memory generalization

**Fig 2:**
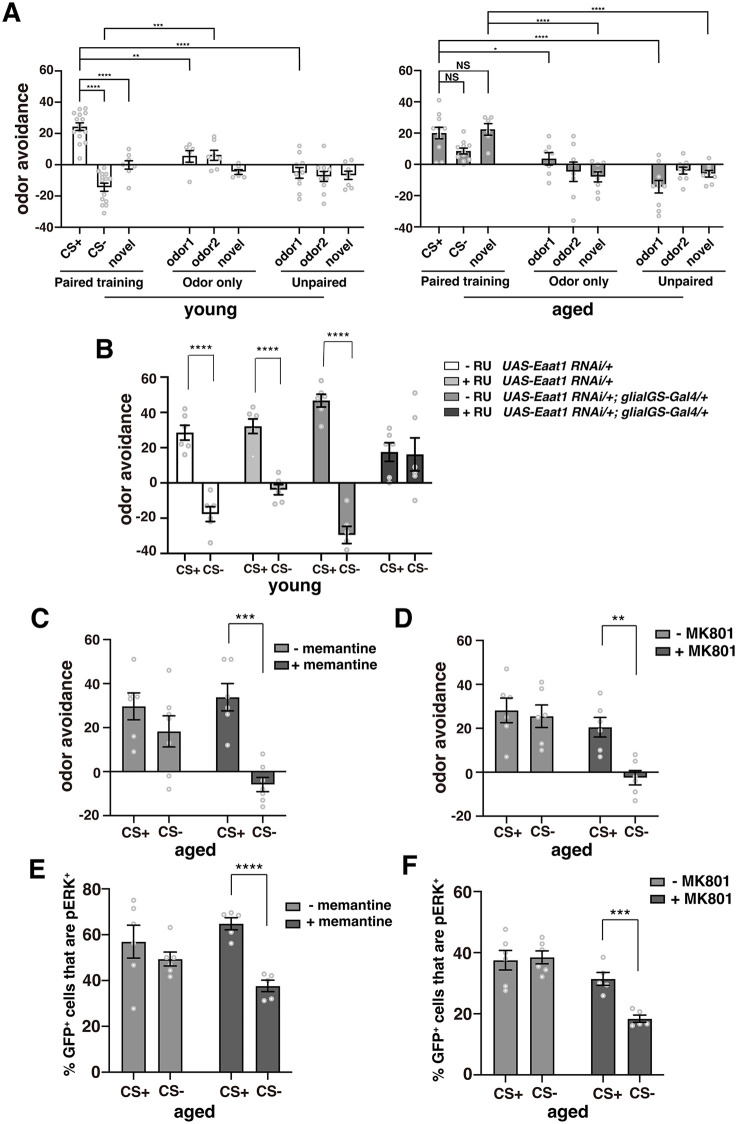
Long-term memory impairments in aged flies are associated with memory generalization and non-specific activation of engram cells. **A)** Avoidance of the CS+, CS−, and a novel odor, IAA, 24 hours after spaced training in young and aged flies. Positive avoidance scores indicate that more flies chose air over the odor while negative avoidance scores indicate that flies chose the odor over air. Spaced training (paired training) induced significant avoidance of the CS+ odor, approach toward the CS− odor, and no change in the response to a novel odor in young flies (*N* = 6–15). Control training included training in the absence of shocks (odor only, *N* = 6–8) and training where shocks were unpaired with either odor (unpaired training, *N* = 8–10). In aged animals, paired training resulted in a significant avoidance of the CS+, but also a significant avoidance of the novel odor compared to odor only and unpaired controls. Aged animals also had a tendency to avoid rather than approach the CS−. *, **, ***, and **** indicate *P* < 0.05, 0.01, 0.001, and 0.0001. **B)** Acute inhibition of Eaat1 induces memory generalization behaviors in young flies. Twenty-four hours after spaced training, control flies (−RU *UAS-eaat1 RNAi/+*, +RU *UAS-eaat1 RNAi/+*, and −RU *UAS-eaat1 RNAi/+; glialGS/+*) avoided the CS+ odor, but not the CS− odor. Inhibition of Eaat1 expression (+RU *UAS-eaat1 RNAi/+; glialGS/+*) caused flies to avoid both the CS+ and CS− odors. *N* = 6 for each condition. **C, D)** Memantine **(C)** or MK801 **(D)** feeding after spaced training reduces avoidance of the CS− odor and memory generalization in aged flies. *N* = 6–7. **E, F)** Feeding aged flies memantine **(E)** or MK801 **(F)** after spaced training reduces reactivation of engram cells by the CS− odor, resulting in significant differences between CS+ and CS− dependent engram reactivation. *N* = 5–6. The data underlying this figure are available in S1 Data.

Our results suggest that a major cause of LTM defects in aged flies may be that aversive memories are recalled in inappropriate situations. In standard aversive odor testing experiments, flies are given a choice between two odors, the shock-paired (CS+) odor, and a non-shock-paired (CS−) odor. In young flies, aversive memory engrams are reactivated by the CS+, causing flies to avoid the CS+ and choose the CS− when given a choice between the two odors. However, if aversive engrams are reactivated by both CS+ and CS− odors in aged flies, flies should try to avoid both odors, leading to a reduction in behavioral scores. To determine whether aged flies show increased avoidance of both appropriate and inappropriate odors after training, we examined whether aged flies displayed an increased avoidance towards the CS+, CS−, and a novel odor after training when given a choice between the testing odor and air instead of a control odor. As seen in the left sections of Figs 2A and S1A, 24 hours after spaced training (paired training), young flies show a strong avoidance of the shock-paired odor, an attraction to the unpaired (CS−) odor [18,19], and a neutral response to novel odors. Control training protocols in which flies were mock trained by exposing them to odors in the absence of electrical shocks (odor-alone training) or where odors and shocks were unpaired resulted in neutral responses. Similar to young flies, old flies showed a strong avoidance of the CS+ odor after paired training. However, instead of an attraction to the CS− odor and a neutral response to a novel odor, they showed an avoidance of these odors (Figs 2A and S1A, right sections). Thus, compared to mock or control training methods, old flies subjected to paired training showed a significant increase in avoidance of both a novel odor and the CS+ odor, and a shift from attraction to avoidance of the CS− odor, when compared to young paired-trained flies. Altogether our results suggest that reactivation of aversive LTM engram cells by both appropriate and inappropriate odors in aged flies is accompanied by increased avoidance of these odors. This indicates that one component of age-related impairments in LTM is memory generalization, where aged flies lose the specificity of a memory.

Flies were aged for 40 days at 18 °C to generate old flies for engram imaging experiments in order to inhibit background, non-training-dependent c-Fos expression, while they were aged for 23–25 days at 25 °C for behavioral experiments. To confirm that both methods of aging induce generalization, we verified that memory generalization occurs in flies aged at 18 °C similarly to those aged at 25 °C (S1B Fig).

Paired training consists of exposing flies to two odors, the CS + , which is paired temporally with electrical shocks, and the CS−, which is presented 45 s after shock cessation. While the CS− may have previously been thought of as a control odor to the CS + , more current studies have determined that the CS− can become associated with learned safety or relief from electric shocks and becomes attractive to flies [18,19] (see also CS− effects in Figs 2A and S1A). The engram cells we identified can be classified as aversive since they are preferentially reactivated by the CS+ odor in young flies and induce aversive behaviors when activated. However, to remove possible confounding effects of CS− memory, we also performed CS+ training in which the CS + was paired to electrical shocks and no CS− was presented (S1C and S1D Fig). CS+ training produced similar numbers of GFP+ engram cells as paired training and these engram cells were reactivated preferentially by the CS+ odor compared to a novel odor in young flies, while they were reactivated similarly by CS+ and the novel odor in old flies. Furthermore, as a control, we performed mock odor-only training. In this situation, engram cells were formed, likely because of the altered context during training [20], but these engram cells were reactivated by odors at significantly lower numbers compared to paired and CS+ training in both young and old flies. Overall, our results indicate that odor-specific engram cells are formed by both paired and CS+ training in young flies, but these engram cells lose their odor specificity upon aging resulting in memory generalization.

One possible explanation for apparent memory generalization in old flies is if spaced-trained old flies are unable to distinguish between different odors. However, we found that old flies are able to form short-term memories, which require the ability to distinguish odors, 24 hours after spaced-training (S2A Fig). We further found that a second type of consolidated memory, anesthesia-resistant memory (ARM), which does not require c-Fos positive engram cells, does not induce memory generalization in old flies (S2B Fig). In addition, generalization occurs after spaced training of old *radish* (*rsh*) mutants, which are specifically impaired for ARM (S2C Fig). Thus, memory generalization seems to be a specific phenomenon affecting the reactivation of LTM engram cells by multiple distinguishable odors.

### Inhibition of glutamate activity during memory consolidation prevents memory generalization and inappropriate reactivation of engrams

How does aging cause an increase in memory generalization? LTM formation requires an increase in amounts of the glial glutamate transporter, EAAT1, which functions to clear synaptic glutamate during memory consolidation [16]. Aged flies are unable to increase *Eaat1* expression after training, while artificial expression of *Eaat1* in glia is able to rescue LTM defects in aged flies [16]. This suggests that inappropriate glutamate activity during memory consolidation may induce generalization, leading to reduced LTM. To test this idea, we examined whether artificial inhibition of *Eaat1* induces memory generalization in young flies. We used a glial geneswitch (GS) driver to acutely knock down *Eaat1* expression in glia in young adult flies and observed memory generalization, similar to what we observe in aged wild-type flies (Fig 2B). In a complementary approach, we also inhibited NMDA-type glutamate receptors after spaced training in aged flies using the pharmacological agents, memantine and MK801, and observed an increase in memory specificity (Fig 2C and 2D). When we examined odor-dependent reactivation of engram cells in aged flies, we found that memantine and MK801 feeding after spaced training reduced activation of engram cells by the CS− odor, restoring the preferential activation of engram cells by the CS+ odor (Fig 2E and 2F). These results suggest that activation of NMDA receptors by glutamate during consolidation causes engram cells to be reactivated by inappropriate odors, leading to memory generalization and LTM defects in aged flies.

### Activation of PPL1 dopaminergic neurons by glutamate during consolidation inhibits LTM

**Fig 3:**
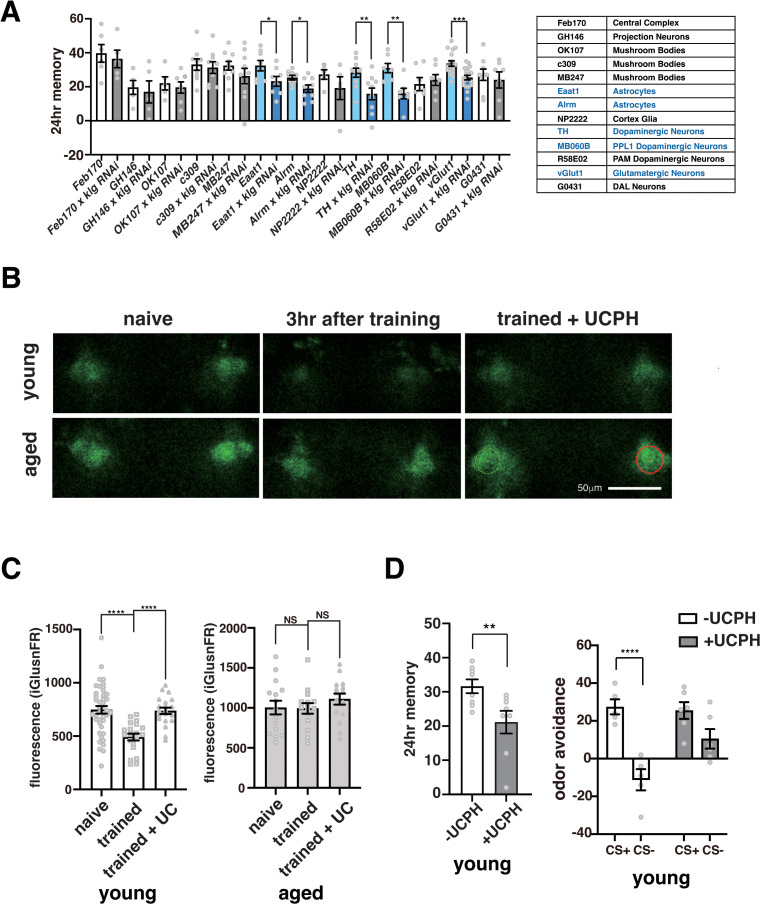
Suppression of Klg in astrocytes, dopaminergic neurons, and glutamatergic neurons inhibits long-term memory. **A)** *klg* was knocked down (*klgRNAi*) in indicated cell types using indicated drivers. Drivers/cell types where *klg* knockdown significantly inhibited LTM are shown in blue. *N* = 5–19. *, **, ***, and **** indicate *P* < 0.05, 0.01, 0.001, and 0.0001. **B)** Representative images of glutamate sensor (iGlusnFR) fluorescence in young and old flies under different conditions. The glutamate sensor, iGlusnFR, was expressed in PPL1 neurons from the MB060B driver and ROIs (green and red circles) were chosen at the α2α′2 compartments of the mushroom bodies. In young flies, glutamate signals were reduced 3 hours after spaced training compared to naïve animals. This reduction was not observed when young flies were fed the EAAT1 blocker, UCPH-101. Reductions in glutamate concentrations 3 hours after spaced training were not observed in aged flies. **C)** Quantification of the data shown in **B)**. *N* = 20–42 for young flies, and *N* = 15 for old flies. **D)** Pharmacological inhibition of EAAT1 activity impairs LTM and induces memory generalization in young flies. Young flies were fed UCPH-101 after spaced training and tested for LTM (right panel) or tested for avoidance of the CS+ and CS− odors at 24 hours (left panel). In the left panel, no significant differences in avoidance of the CS+ and CS− odors were found in flies fed UCPH-101. Right panel, *N* = 8, Left panel, *N* = 5–6. The data underlying this figure are available in S1 Data.

Where does glutamate signaling need to be inhibited to prevent memory generalization? Upregulation of *Eaat1* in glial cells after spaced training requires neuron/glia interactions mediated by the homophilic cell adhesion molecule, Klg [16]. Thus, to identify candidate neurons and glia involved in LTM and memory generalization, we knocked down *klg* in various cell types and searched for reductions in LTM (Fig 3A). We found that knocking down *klg* in the ellipsoid bodies and mushroom bodies had no significant effects on memory. In contrast, knocking down *klg* in astrocyte-glia, glutamatergic neurons, and dopaminergic neurons significantly reduced LTM. This suggested that EAAT1 needs to inhibit glutamate signaling between presynaptic glutamate neurons and postsynaptic dopaminergic neurons during LTM consolidation to prevent memory generalization. We further found that knocking down *klg* specifically in the PPL1 subset of dopaminergic neurons using the MB060B driver inhibited LTM, while knocking down *klg* in the PAM cluster dopaminergic neurons did not (Fig 3A).

The MB060B driver labels a subset of PPL1 neurons that are proposed to regulate consolidation and forgetting of LTM [21,22]. To verify that glutamate input to these neurons is inhibited during memory consolidation, we expressed the fluorescent glutamate sensor, iGlusnFR, in these neurons to measure extracellular glutamate after spaced training [23,24]. Compared to naïve flies, we measured a significant reduction in glutamate 3 hours after spaced training in young flies (Fig 3B and 3C), which correlates with an increase in *Eaat1* expression after training (S3A Fig). This result is consistent with previous studies [16] and suggests that increased *Eaat1* expression during consolidation decreases glutamatergic activation of PPL1 neurons. When we fed young flies a glutamate transporter blocker, UCPH-101, after training, we found that the reduction in glutamate at 3 hours was abolished, LTM was impaired, and avoidance of the CS− odor was increased (Fig 3B–3D). This indicates that glutamate transporter activity is required to reduce glutamate during consolidation, and a failure to do this results in LTM impairment and memory generalization. In contrast to young flies, we did not observe a decrease in extracellular glutamate after spaced training in aged flies. This result is consistent with the inability of aged flies to increase EAAT1 expression after spaced training [16] and prevent memory generalization.

### Glutamate activation induces low-frequency activity in PPL1 neurons

**Fig 4:**
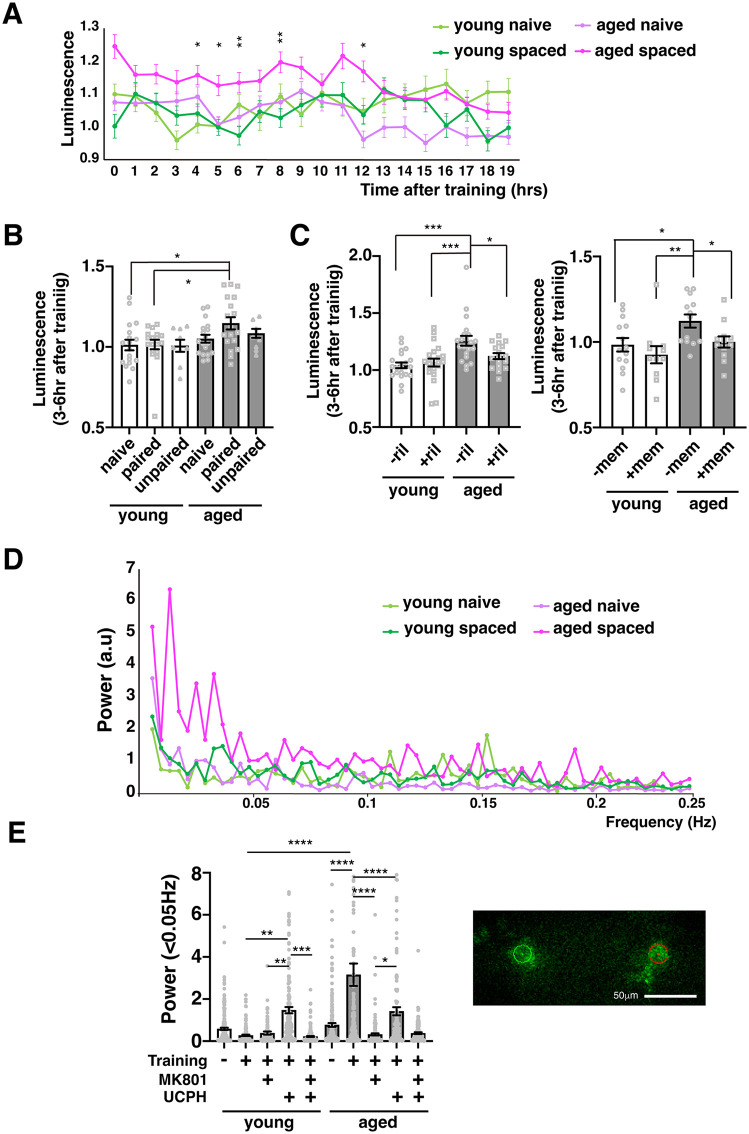
Activity of PPL1 dopaminergic neurons increases after spaced training in aged flies. **A)** Activity of PPL1 DANs was measured in young and aged, naïve and trained *MB060B>FLP, Lola(FRT)stop(FRT)Luc* flies (*N* = 18 for each condition). No significant differences were observed in luciferase activity between young and aged naïve flies or between young naïve and young trained flies. Asterisks indicate significant differences between aged trained vs. young trained flies. *, **, ***, and **** indicate *P* < 0.05, 0.01, 0.001, and 0.0001. **B)** Luminescent activity in indicated flies 3–6 hours after spaced training. Activity was normalized to that of young naïve flies. *N* = 9–18. **C)** Effects of riluzole and memantine feeding on PPL1-DAN activity. *N* = 17–22 for riluzole experiments and *N* = 11–14 for memantine experiments. **D)** Power spectrum in PPL1 neurons of young and aged, naïve and spaced trained flies detected using GCaMP. **E)** Effects of the NMDAR inhibitor, MK801, and the EAAT1 inhibitor, UCPH-101 on power at frequencies <0.05 Hz. *N* = 9–14. The inset is a representative image of the α2α′2 compartment region of the mushroom bodies and ROIs where GCaMP fluorescence was measured in PPL1 neurons. The data underlying this figure are available in S1 Data.

To next determine how reduced glutamate signaling affects PPL1 dopaminergic neuronal activity during memory consolidation, we examined the activity of PPL1 neurons using a bioluminescence-based neural activity reporter. Longitudinals lacking (Lola) is a transcription factor whose expression is regulated by neuronal activity, and Lola promoter luciferase constructs have been used to measure neuronal activity [25–27]. We used flies expressing Lola_p_-luciferase in PPL1 neurons to measure the activity of these neurons after spaced training. In young flies, PPL1 activity after training was similar to activity in naïve and unpaired control flies (Fig 4A and 4B). In contrast, in aged flies, PPL1 activity was significantly increased between 3 and 6 hours after spaced training (Fig 4A and 4B). Administration of riluzole, a glutamate receptor inhibitor [28], or memantine, an NMDA receptor antagonist (Fig 4C), abolished this increase, suggesting that it is caused by excess glutamate activity. Altogether, our data are consistent with a model where glutamate activity needs to be reduced during consolidation to prevent memory generalization. In young flies, this is accomplished by increasing glial EAAT1 expression. However, aged flies are unable to increase EAAT expression resulting in increased PPL1 activity during consolidation.

To verify that spaced training increases PPL1 activity after training in aged flies, we next examined PPL1 activity using calcium imaging. We observed a significant increase in Ca^2+^ oscillations at frequencies below 0.05 Hz 3 hours after spaced training in aged flies that was not present in young flies (Fig 4D and 4E). Application of

the NMDA receptor antagonist, MK801, to aged flies reduced PPL1 activity to the levelof young flies (Fig 4E), suggesting that increased glutamate signaling to NMDA receptors is responsible for increased PPL1 activity in aged flies. Furthermore, feeding the Glu transporter blocker UCPH-101 after training caused an increase in Ca^2+^ oscillations below 0.05 Hz in young flies, suggesting that impaired Glu uptake after training is responsible for the increase in Ca^2+^ oscillations in aged flies. UCPH-101 feeding might also be expected to increase Ca^2+^ oscillations in old flies, but instead we observed a decrease, possibly indicating that further increases in glutamate activity above the increase already found in old flies may induce glutamate toxicity [29].

Neuronal activity increases phosphorylation of ribosomal protein S6, and phospho-S6 can be used to identify active neurons [30]. We examined phosphorylated S6 in TH+ neurons and found that spaced training increases phospho-S6 signals in aged, but not young, flies (S3B and S3E Fig). This increase does not occur after massed training (S3C Fig), supporting the idea that spaced training specifically increases dopaminergic activity in aged flies. Ectopic expression of the glutamate transporter, *eaat1*, in glia reduced phospho-S6 signals in dopaminergic neurons after spaced training in aged flies (S3D Fig), suggesting that reductions in Eaat1 are responsible for increased dopaminergic activity and impaired LTM in aged flies.

### NMDAR-dependent activation of PPL1 neurons regulates memory generalization

**Fig 5:**
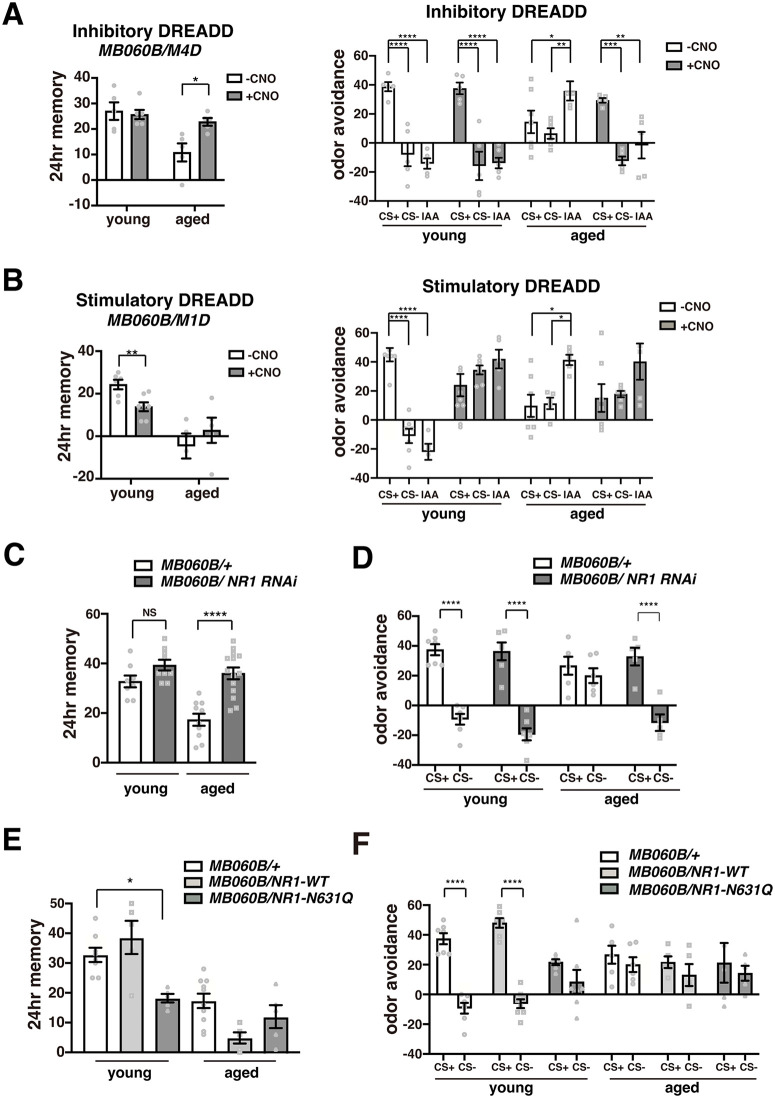
PPL1 activity regulates memory generalization. **A)** Effects of inhibition of PPL1 neurons on LTM scores (left panel) and generalization (right panel) in young and aged flies. Inhibitory DREADDs were activated after spaced training by feeding flies CNO. *N* = 5–7. *, **, ***, and **** indicate *P* < 0.05, 0.01, 0.001, and 0.0001. **B)** Effects of stimulation of PPL1 neurons on LTM scores (left panel) and generalization (right panel) in young and aged flies. Stimulatory DREADDs were activated as described in **A)**. *N* = 5–10. **C, D)** Knocking down NR1 in PPL1 neurons rescues LTM defects **(C)** and suppresses memory generalization **(D)** in aged flies. *N* = 8–14 for LTM experiments, and *N* = 5–7 for generalization experiments. **E, F)** Overexpression of Mg^2+^ block-deficient NR1 in PPL1 neurons inhibits LTM **(E)** and induces memory generalization **(F)** in young flies. *N* = 5–10 for each condition. The data underlying this figure are available in S1 Data.

Our results indicate that aberrant activation of PPL1 neurons during consolidation causes memory generalization in aged flies. If this is the case, artificial activation of PPL1 neurons in young flies should induce generalization, while inhibiting these neurons in aged flies should suppress generalization. Thus, we examined whether altering PPL1 activity after spaced training using the DREADD system [31,32] affected LTM and generalization. When we activated the inhibitory DREADD, hM4Di, in PPL1 neurons by feeding *MB060B>hM4D*i flies CNO after training, we observed no change in LTM or generalization in young flies, while we observed a restoration of LTM (Fig 5A, left panel) and a reduction of generalization in aged flies (Fig 5A, right panel). Conversely, when we activated the stimulatory hM1Dq DREADD in PPL1 neurons after training, we observed a significant decrease in LTM and an increase in generalization in young flies, but no change in memory or generalization in aged flies (Fig 5B). Thus, LTM impairment and increased generalization can be caused by aberrant activation of PPL1 neurons in young flies, while these defects can be rescued by inhibition of PPL1 in aged flies.

To verify that glutamate regulates PPL1 activity through NMDARs, we next manipulated NMDARs on PPL1 neurons and examined effects on LTM and generalization. We found that decreasing NMDAR expression using RNAi in PPL1 neurons rescued LTM defects and reduced generalization in aged flies without affecting memory or generalization in young flies (Figs 5C, 5D, and S4A). Notably, the converse experiment, overexpression of wild-type NMDARs did not inhibit LTM or induce generalization in young flies. Activation of NMDARs requires two steps, (1) binding of extracellular glutamate and (2) depolarization to remove Mg^2+^ ions that block channel conductance, suggesting that overexpression of NMDARs on its own is insufficient to increase glutamate-dependent activation of PPL1 neurons. When we overexpressed an NMDAR Mg^2+^ block mutant, which can be activated by glutamate in the absence of depolarization (NR1 N631Q), we observed a reduction in LTM and an increase in generalization in young flies (Figs 5E, 5F, and S4A). This suggests that age-related impairments in LTM occur through aberrant activation of NMDARs through a combination of two mechanisms, increased glutamate signaling and removal of Mg^2+^ block. One possible mechanism for removing Mg^2+^ block at old ages would be an age-dependent reduction in extracellular Mg^2+^ concentration. However, when we measured the extracellular concentration of various cations at young and old age, we did not observe any decrease in Mg^2+^ upon aging (S4B Fig). Thus, a different mechanism, such as increased depolarization of PPL1 neurons, occurs upon aging to contribute to memory reductions and increased generalization.

### Knockdown of Dop2 receptors in engram cells reduces generalization in aged flies

**Fig 6:**
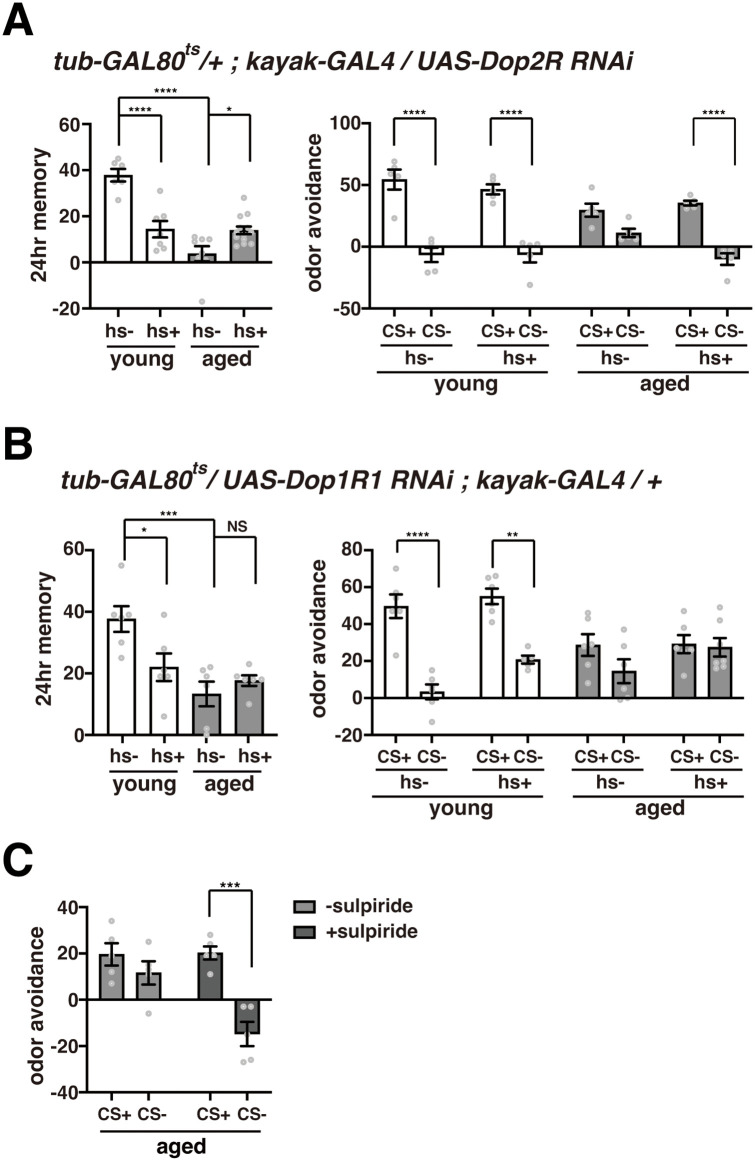
Inhibition of Dop2R reduces memory generalization. **A)** Left panel, LTM scores of young and old *tub-GAL80*^*ts*^*/+; kayak-GAL4/UAS-Dop2RRNAi* flies trained at GAL80^ts^ permissive (-hs) or restrictive (+hs) temperatures. Temperature shifts for +hs flies are described in Fig 1A. Knocking down *Dop2R* improves LTM, specifically in old flies. *N* = 6–13. Right panel, avoidance of the CS+ and CS− odors in these flies. Knocking down *Dop2R* inhibits memory generalization in old flies. *N* = 5. *, **, ***, and **** indicate *P* < 0.05, 0.01, 0.001, and 0.0001. **B)** Left panel, LTM scores of young and old *tub-GAL80*^*ts*^*/UAS-Dop1R1RNAi;kayak-GAL4/+* flies trained at GAL80^ts^ permissive (−hs) or restrictive (+hs) temperatures. Knocking down *Dop1R1* does not affect LTM in old flies. *N* = 6–14. Right panel, avoidance of the CS+ and CS− odors in these flies. Knocking down *Dop1R1* does not affect memory generalization in old flies. *N* = 5. **C)** Sulpiride feeding restores memory specificity in old flies. 5 μM sulpiride or vehicle was fed to flies from the end of training until testing, and avoidance of the CS+ and CS− odors was measured. *N* = 5. The data underlying this figure are available in S1 Data.

Altogether, our data suggest that dopamine released from PPL1 neurons during memory consolidation alters plasticity such that memory engram cells are activated by a variety of odor-responsive neurons. To next determine whether dopamine may be acting directly on engram cells to alter their activity, we knocked down dopamine receptor subtypes (Dop1R1, Dop1R2, Dop2R, and DopEcR) in kayak-positive engram cells at Gal80^ts^ restrictive temperatures using *tub-Gal80*^*ts*^*/UAS-Dop1R1RNAi;kayak-Gal4/+, tub-Gal80*^*ts*^*/+;kayak-Gal4/UAS-Dop1R2RNAi, tub-Gal80*^*ts*^*/+;kayak-Gal4/UAS-Dop2RRNAi* and *tub-Gal80*^*ts*^*/+;kayak-Gal4/UAS-DopEcRRNAi* flies. We raised these flies at 18 ℃ where Gal80^ts^ prevents knockdown, and then shifted them to 30 ℃ to knock down dopamine receptors during spaced training and memory consolidation. Knocking down Dop1R1, Dop1R2, and Dop2R receptors inhibited long-term memory in young flies, while knocking down DopEcR had no significant effects (left panels of Figs 6A, 6B, S5A, and S5B). Knocking down Dop1R1, Dop1R2, and DopEcR did not affect the already impaired LTM of old flies, while knocking down Dop2R improved LTM (left panels of Figs 6A, 6B, S5A, and S5B). Furthermore, while knocking down the other DopRs had no effects on generalization in either young or old flies, knocking down Dop2R prevented memory generalization in old flies without affecting memory specificity in young flies (right panels of Figs 6A, 6B, S5A, and S5B). These data are consistent with a model where dopamine release from PPL1 neurons acts on Dop2Rs on engram cells to induce memory generalization. Also consistent with this model, we found that feeding flies the Dop2R antagonist sulpiride during memory consolidation also prevented memory generalization in old flies (Fig 6C).

### Correlate to memory generalization visualized in α2sc-MB output neurons

**Fig 7:**
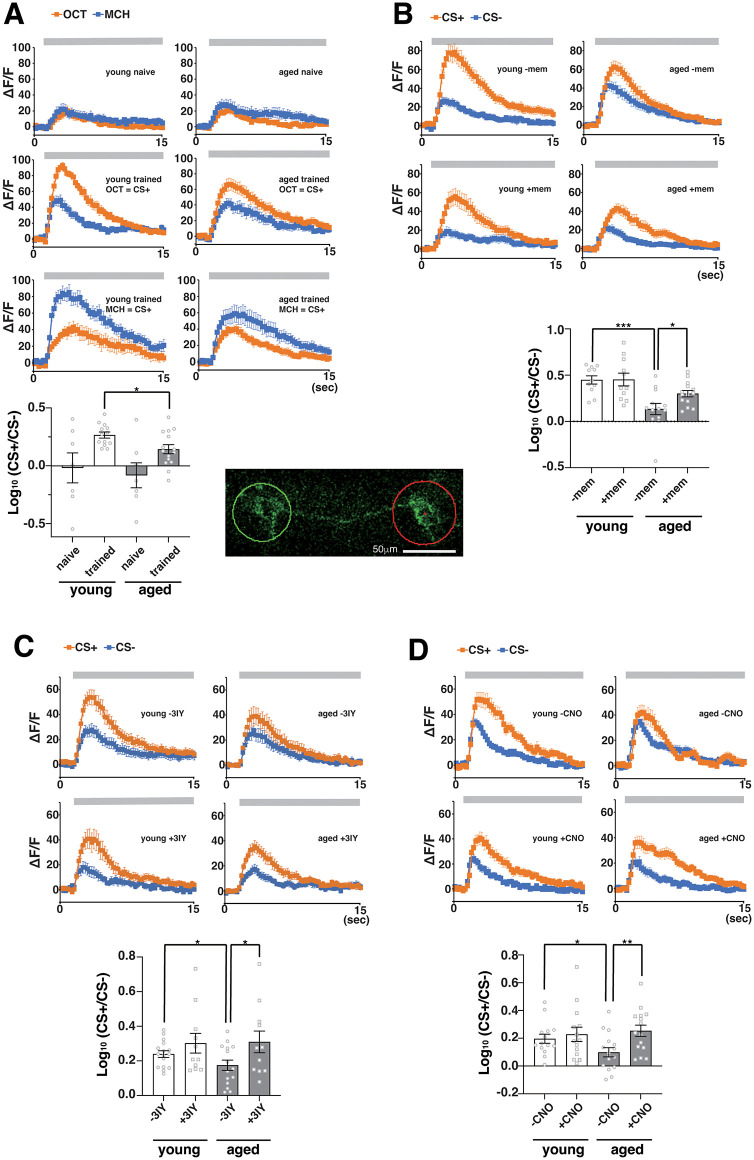
Training-dependent odor responses in α2sc-MBON are altered in aged flies. **A)** Upper panels, Ca^2+^ responses in α2sc-MBONs after exposure to indicated odors measured in naïve and spaced-trained, young and aged flies. The gray bar above each graph indicates the duration of odor exposure. Lower panel, quantification of the log_10_ of the CS+/CS− ratio of the data in the upper panels. In naïve animals, the CS+/CS− ratio refers to the odor 1/odor 2 ratio (the odors that would have been the CS+ and CS− odors if the animals had been trained). *N* = 6–8. *, **, and *** indicate *P* < 0.05, 0.01, and 0.001. Photo insert: a representative image of the α2sc-MBON fluorescence ROI at the mushroom body α2α′2 compartment region. **B)** Memantine feeding after training increases response differences to the CS+ and CS− odors in aged flies. *N* = 11–14. **C)** 3-iodo-tyrosine feeding after training increases response differences to the CS+ and CS− odors in aged flies. *N* = 11–15. **D)** DREADD-dependent suppression of PPL1 activity after training also increases response differences to the CS+ and CS− odors in aged flies. *N* = 14–16. The data underlying this figure are available in S1 Data.

PPL1 dopaminergic neurons regulate the plasticity between MB Kenyon cells and MB output neurons, including α2sc MB output neurons (α2sc-MBONs), which are required for retrieval of LTM [33,34]. To next determine how age-dependent changes in PPL1 activity translate to changes in MB output, we examined activity in α2sc-MBONs in naïve and spaced-trained animals. When we measured activity during odor exposure in naïve animals, we observed similar Ca^2+^ responses to both Oct and MCH odors at young and old ages with no significant differences between responses to each odor (Fig 7A). Twenty-four hours after spaced training of young flies, we observed significantly higher Ca^2+^ responses to the CS+ odor compared to the CS− and to novel odors (Figs 7A and S6). In aged flies, this difference was reduced such that the log_10_ ratio of CS+ to other odors was significantly lower in old flies compared to young flies. To determine whether this reduction in odor differential may be related to memory generalization, we examined whether MBON-α2sc responses could be restored in aged flies by inhibiting NMDAR signaling after spaced training. We examined MBON-α2sc responses in flies after memantine feeding and observed a significant increase in the log ratio of CS+ to CS− responses in old, but not young, flies (Fig 7B). We further found that inhibiting dopamine production in aged flies by feeding them the tyrosine hydroxylase inhibitor 3-iodotyrosine (3IY) also restored odor-specific differences in old flies (Fig 7C). Similarly, inhibiting PPL1 activity using DREADD restored appropriate a2sc responses (Fig 7D). Altogether, our data suggest that glutamate signaling onto PPL1 dopaminergic neurons during memory consolidation in aged flies alters plasticity within the MBs such that other odors besides the CS+ induce avoidance behaviors. The ability of other odors to induce avoidance behaviors is reflected by a decrease in the differential response of α2sc-MBONs to CS+ versus other odors.

## Discussion

Here, we determined that increased memory generalization is a major cause of age-related impairments in LTM in *Drosophila*. At the cellular level, we find that memory engram cells in old flies are reactivated by inappropriate cues, leading to activation of memory-associated avoidance behaviors. Generalization depends on activity of PPL1 dopaminergic neurons and activation of D2 dopaminergic receptors on engram cells during memory consolidation. Further upstream, we find that glutamate acts upon NMDA receptors on PPL1 neurons to regulate dopaminergic activity. Overall, our results suggest that glutamatergic activation of PPL1 neurons needs to be reduced during consolidation to maintain memory specificity and prevent inappropriate generalization.

Various studies have demonstrated that glial transcription and translation are essential for LTM [15,35–38]. In flies, we previously showed that spaced training increases activity of the glial transcription factor Repo, which in turn induces expression of *Eaat1* to decrease glutamate signaling [15,16]. Activation of Repo depends on the homophilic cell adhesion molecule, Klg, which mediates interactions between neurons and glia [17]. In aged flies, expression of *klg*, *repo*, and *Eaat1* is reduced, and LTM is impaired [16]. Importantly, LTM deficits in old flies can be rescued by ectopic expression of *klg*, *repo*, or *Eaat1*, while inhibition of any of these components reduces LTM in young flies. Our current work identifies the cellular and molecular pathways downstream of EAAT1 to explain how glutamate signaling affects PPL1 dopaminergic signaling to influence engram cell reactivation and memory generalization. Altogether, our findings support a model (S7 Fig) that explains how aberrant generalization is prevented in young flies, and how dysfunction of this pathway induces generalization in old flies.

In *Drosophila*, odor information is transmitted to the MBs via projection neurons from the antennal lobes which activate a sparse subset of MB Kenyon cells [39]. Odor specificity is maintained because different odors activate different sparse subsets of Kenyon cells. However, the connection between odor-responsive cells and memory engram cells has been unclear. If odor-responsive Kenyon cells are directly converted into engram cells by spaced training, a large increase in engram cells should occur upon generalization. However, we did not observe a significant increase in engram cell numbers in aged versus young flies. Aged flies can distinguish between different odors, they can learn associations [2], and they form normal ARM [7], suggesting that the sparse subsets of MB Kenyon cells responding to individual odors are maintained in aged flies. Thus, our data is consistent with a model where engram cells are formed downstream of odor-responsive Kenyon cells. Specific memories are formed by specific increases in connectivity between upstream odor-responsive neurons and downstream engram cells, while generalization is likely caused by non-specific increases. Consistent with our contention that odor-responsive Kenyon cells and engram-type Kenyon cells consist of distinct populations, MB engram cells are found in α/β type Kenyon cells which innervate the α/β lobes [12], while odor-responsive Kenyon cells are not restricted to this cell type [40]. Further, the number of synaptic sites has been shown to increase significantly in old flies [41], suggesting that large non-specific increases in synaptic connectivity occur upon aging.

Supporting the idea that PPL1 neurons regulate memory generalization, activation of PPL1 neurons during consolidation using the DREADD system induced generalization and inhibited LTM in young flies. This effect was not observed in old flies suggesting that the generalization already present in old flies is caused by increased PPL1 activity and occludes further effects. Conversely, artificial inhibition of PPL1 neurons during consolidation prevented generalization and rescued LTM in aged, but not young, flies. Mushroom body Kenyon cells extend axons into different MB lobes (γ, α/β, and α′/β′) which are further separated into 11 different compartments that are innervated by different dopaminergic neurons and that innervate different MB output neurons [42]. The MB060B driver we used in our studies drives expression in four classes of PPL1 dopaminergic neurons innervating the γ2α′1, α′3, α′2α2, and α3 mushroom body compartments [42]. Previous studies have reported roles of these or subsets of these neurons in regulating forgetting [43], gating the formation of LTM [22], and sleep [44]. In particular, dopaminergic neurons innervating γ2α′1 and α′2α2 have been shown to display spontaneous low-frequency (0.05–0.10 Hz) activity [22,43], consistent with our data demonstrating that increased activity of these neurons increases low-frequency power.

How does the function of dopamine in memory generalization relate to its other roles in the mushroom bodies? Dopamine has multiple functions in the mushroom bodies. For example, artificial activation of dopaminergic neurons can bypass the requirement for electrical shocks [the unconditioned stimulus (US)] during aversive memory formation [45–49]. This has led to the proposal that dopamine may either convey US information to the mushroom bodies or function as a strong reinforcer of neural plasticity. In contrast, other studies, including ours, have demonstrated that dopamine also has deleterious effects on memory. Artificial activation of PPL1 neurons after training inhibits anesthesia-resistant memory [22], and formation of LTM has been shown to require neuropeptide F, which inhibits activity of PPL1 neurons, during memory consolidation [50]. Furthermore, activity of PPL1 dopamine neurons acting through Dop1R2 receptors has been shown to accelerate forgetting of short-term memories and ARM [21] and decrease sleep [51]. While it is uncertain how these different functions of dopamine can coexist without interference, they seem to be associated with different spatial and temporal release patterns. Thus, dopamine may be required at early time points during memory formation, but its effects at later time points are more nuanced. In addition, our results indicate that activity of D2-type dopamine receptors rather than D1-type receptors, increases non-specific engram activation. During periods of memory consolidation, sleep increases, and memory-specific pathways are reactivated. Activity of D2 receptors may interfere with this reactivation, shifting plasticity from specific to non-specific pathways. Consistent with this idea, we find that differential activation of the α2sc MBON by the shock-paired odor compared to non-shocked odors is reduced in old flies.

Work from others groups suggest that aspects of our identified generalization pathway may be conserved in mammals and may be altered by aging and in diseases such as PTSD, severe depression and anxiety [3–6]. Generalization of semantic memories has been shown to increase during healthy aging in humans [52,53], and generalization of fear memories increases in old rats [54]. Generalization is regulated by dopamine activity [55], particularly D2 receptor activity in humans and other mammals [1]. Further, activity of NMDA-type glutamate receptors has been implicated upstream of dopamine in regulating activity of traumatic fear memories, which have been associated with increased generalization [56,57]. Altogether, these data suggest that activation of D2 receptors on engram cells during memory consolidation induces memory generalization, while inhibition of these receptors prevents it.

## Materials and methods

### Fly stocks and genetics

Flies were raised under a 12 h: 12 h, light: dark cycle at 25 °C and 60% humidity. All flies used in this study were outcrossed to our wild-type line, w(CS10), for at least six generations to normalize genetic backgrounds. For aging, approximately 100 flies of mixed sex were raised in food vials and transferred to fresh vials every 2 or 3 days. Young flies consisted of flies raised for 3−5 days after eclosion at either 25 or 18 °C (to match aged flies), while aged flies consisted of flies raised for 23−25 days at 25 °C or 40 days at 18 °C. *UAS-mCD8-GFP; kayak-GAL4/Tub-GAL80*^*ts*^, and *Tub-GAL80*^*ts*^*/+; kayak-GAL4/UAS-Dop receptor RNAi* flies were aged at 18 °C to inhibit background, training-independent *kayak-Gal4* expression, while flies that did not contain *Tub-GAL80*^*ts*^ were aged at 25 °C. *UAS-mCD8-GFP; kayak-GAL4/Tub-GAL80*^*ts*^, *UAS-ChR2-YFP; kayak-GAL4/Tub-GAL80*^*ts*^, *UAS-NR1*^*+*^ and *UAS-NR1*^*N631Q*^ [12,58], *UAS-Eaat1-RNAi* and *EAAT1-GAL4* [29], *UAS-Eaat1* (Bloomington#8202), the glial-geneswitch line, GSG3285-1, *UAS-klg-RNAi* (*klg*^*36162*^) [15] have all been described previously. *Feb170* was a gift from T. Tully [59], and *Alrm-GAL4* was a gift from M. Freeman [60], *NP2222* was a gift from T. Awasaki [61], *UAS-FLP; lola>stop>Luc* was a gift from K. Keleman [26], and *UAS-hM4Di*, *UAS-hM1Dq* were gifts from C. D. Nichols [31]. *GH146* (#30026), *c309* (#6906), *MB247* (#50472), *TH-GAL4* (#8848), *R58E02* (#41347), *G0431* (#12023), *UAS-iGlusnFR* (#59611), *UAS-GCaMP3* (#32116), *UAS-NR1RNAi* (#41667), *rsh^1^* (#79209), *vGlut1-GAL4* (#24635), *R34B02-lexA* (#53631), *lexAop-GCaMP6f* (#44277), *UAS-Dop2R* *RNAi* (#78804), *UAS-DopEcR RNAi* (#31981), *Tub-GAL80^ts^* (#7108), and *UAS-Dop1R2* *RNAi* (#26018) were obtained from the Bloomington Stock Center (Indiana University), *MB060B* and *MB080c* (MBON-α2sc, splitGAL4) were obtained from the Janelia Fly Facility, *OK107* (#106098) was obtained from the Kyoto Drosophila Stock Center, and *Dop1R1* *RNAi* (#107058KK) was obtained from the Vienna Drosophila Resource Center.

### Learning and memory assays

Standard single-cycle conditioning was performed as previously described [62]. Approximately 100 flies were first exposed for 1 min to the CS+ odor, which was paired with the aversive US, 12 pulses (1.5 s duration each) of 60 V DC electric shocks. After washing out the CS+ odor with air for 45 s, flies were next exposed for 1 min to a CS− odor, which was not paired to the US. For CS+ and CS− odors, a 1:2,000 fold dilution of 3-octanol (OCT) in mineral oil and a 1:1,000 fold dilution of 4-methylcyclohexanol (MCH) in mineral oil were used alternately. Isoamylacetate (IAA) (1:1,000 in mineral oil) and benzaldehyde (BEN) (1:4,000 in mineral oil) were used as novel odors for paired training studies, while OCT or MCH were used as novel odors for CS+ training studies. For standard testing of learning and memory, flies were given a choice between the CS+ and CS− odors in a T maze. A performance index (PI) was calculated as (% of flies that chose the CS− odor) − (% of flies that chose the CS+). Spaced and massed training sessions were also performed as described previously [11]. Spaced training consists of 10 single-cycle training sessions, with 15 min rest intervals between trainings. Massed training consists of 10 cycles of training without any rest intervals. After training, flies were stored in an 18 °C incubator on a 12 h:12 h, light: dark cycle until testing. Testing was performed 24 hours after spaced or massed training.

### Odor avoidance

To measure avoidance of individual odors after training, an odor was delivered to one side of a T maze while air was delivered to the other side. An avoidance index (AI) was calculated as (% of flies that chose air) − (% of flies that chose the odor). MCH, OCT, BEN, and IAA were used as test odors. Odor avoidance of flies trained using paired training was compared to two controls, flies trained using an odor-only protocol where flies were exposed to odors in the absence of electrical shocks, and flies trained using an unpaired protocol where electric shocks were delivered 2 min before exposure to the first odor.

### Drug treatments

For RU486 treatment, RU486 (mifepristone, Sigma) was dissolved in ethanol, and mixed with fly food to a final concentration of 1% ethanol and 0.5 mM RU. For riluzole (1 mM, Sigma), memantine (20 ug/ml, Sigma), MK801 (0.1 mg/ml, TOCRIS), UCPH-101 (100 uM, abcam), 3IY (5 mg/ml, TCI), and sulpiride (5 uM, Sigma) treatments, flies were transferred to vials containing strips of Whatman filter paper soaked with indicated concentrations of each drug and 5% sucrose. RU486 was fed to flies from 2 days prior to training until testing and UCPH-101, riluzole, memantine, 3IY, and sulpiride were fed to flies after spaced training until either testing or imaging.

### Optogenetics

For light stimulation, blue and green LED light strips (JW-system co., type 5050) were wrapped around each arm of a *Drosophila* T-maze. Flies were allowed to choose between blue and green light for 90 s in an otherwise dark 18 °C room. 0.15 μM all-*trans-*retinal (ATR, Sigma) was mixed in *Drosophila* food and fed to UAS-ChR2-YFP; Kayak-Gal4/Tub-GAL80^ts^ flies for 24 hours before testing.

### Chemogenetics

DREADD transgenic flies were starved 3 hours before training and fed 3 mM CNO (TOCRIS) in 5% Sucrose after training. Control flies were fed 5% Sucrose without CNO.

### Histology

For immunohistochemistry of engram cells, fly heads were fixed in 4% paraformaldehyde, 15% picric acid in 0.1M phosphate buffer for 2 hours on ice, and brains were dissected. For immunohistochemistry of activated dopaminergic neurons, 10 μm frontal paraffin sections of heads were cut and processed as previously described [63,64]. Antigens in paraffin sections were reactivated by heating for 10 min at 95 °C in 10 mM sodium citrate, pH 6.0. For immunostaining of activated MB060B neurons, fly heads were fixed in 4% paraformaldehyde, and antigens were reactivated by heating at 70 °C in 10 mM sodium citrate for 10 min. Primary antibodies used in this study were chick anti-GFP (1:400, abcam #13970), rabbit anti-phosphop44/42 MAPK (1:400, Cell Signaling #4370), Rabbit anti-phosphoS6 ribosomal protein 240/244 (1:200, Cell signaling, Cat#2215), and mouse monoclonal anti-TH (1:200, Immunostar, Cat#22941). All secondary antibodies (Alexa555, Alexa488 conjugated) were used at 1:400 dilutions. Fluorescence images in fly brains were obtained using a confocal laser microscope (SP-8, Leica or FV1000, Olympus).

### Cation concentrations in hemolymph

Double-barreled ion-sensitive microelectrodes for recording the extracellular concentration of [Mg^2+^]_e_ and [Ca^2+^]_e_ [K^+^]_e_ and [Na+]_e_, or [Ca^2+^]_e_ were inserted into the head hemolymph of immobilized tethered flies. The reference barrel was filled with 154 mM NaCl solution, the ion-sensitive barrel with the ionophore cocktail (Sigma, Magnesium Ionophore II 63083; Sigma, Calcium Ionophore I 21048) and 100 mM MgCl_2_ or 100 mM CaCl_2_. The ion-sensitive microelectrodes were calibrated before measurements in the specimen [65]. As magnesium ionophore cocktails also measure Ca^2+^ with almost the same sensitivity as Mg^2+^, we subtracted the [Ca^2+^]_e_ from the measured [Mg^2+^]_e_.

### In vivo imaging

Imaging of calcium responses in α2scMBON was performed in endogenous hemolymph 20–24 hours after spaced training. Flies were immobilized on ice and mounted in an imaging chamber, allowing free movement of the antenna and legs. The head cuticle was removed, the exposed brain was covered with UV glue (Norland Products), and the fly was placed under a confocal microscope (A1R, Nikon). Microscope conditions for in vivo calcium imaging were previously published [66]. Flies were sequentially exposed to the CS+ followed by the CS−, or the CS− followed by the CS+, or a third odor IAA followed by the CS+ for 30 s/odor at an airflow of 200 ml/min. Exposure to each odor was followed by a 2 -min wash out with air.

For imaging both calcium responses (GCaMP) and glutamate measurements (iGlusn FR) in DANs after spaced training, the head cuticle was removed at indicated time points, and exposed brains were covered in HL3 medium (in mM, NaCl, 70; sucrose, 115; KCl, 5; MgCl_2_, 20; Ca Cl_2_, 1.5; NaHCO_3_, 10; trehalose, 5; Hepes, 5; pH 7.3). For the experiment described in Fig. 4E, indicated flies were fed 100 uM UCPH-101 immediately after training for 3 hours prior to imaging, while 0.1 mg/ml MK801 was applied directly to the HL3 medium bathing exposed fly brains using a peristaltic pump. Imaging was performed under a confocal microscope (A1R, Nikon) with the objective positioned above the head of the fly such that the vertical lobes of the mushroom bodies were located along the z-axis. GCaMP and iGlusn FR signals within the mushroom body vertical lobes were chosen as regions of interest and correspond to signals from MB1 and MP1 dopaminergic neurons. Fluorescent images were captured at a frequency of 4 Hz and *F*_0_ was calculated as the mean fluorescent intensity of the 5 frames immediately preceding odor presentation. Δ*F*/*F*_0_ values were calculated using this baseline. To compare responses to the CS+ odor to responses from a second odor B (the CS− odor or a novel odor) within the same animal, we calculated log-transformed response ratios (log_10_(peak Δ*F*/*F*_0_ for the CS+)/(peak Δ*F*/*F*_0_ for odor B). This metric reflects odor selectivity as a fold difference, yields a symmetric distribution around zero, and avoids the instability of percentage-based measures when baseline responses are small. For pharmacological treatments, agents were diluted in HL3 medium and applied directly to the brain by micropipette. For power spectrum studies, calcium images were recorded for 4 min 16 s at 4 Hz in naïve animals and 3 hours after spaced training in trained animals. Fourier transformations of 1,024 frames were calculated using Excel, and power frequency graphs were generated.

### Luminescence assay

The luminescence assay for detecting neural activity in freely moving adult flies was monitored as previously described [26]. Flies were starved for 5 hours and fed 40 mM D-Luciferin (GOLDBIO) in a 5% sucrose solution overnight prior to training. For luminescence measurements after training, flies were placed into 96-well plates with 40 mM D-Luciferin in a 5% sucrose solution. Luminescence was measured every 16 min over 20 hours. Relative luminescence was calculated by dividing the luminescence of the experimental group by the luminescence of the genetic controls at each time point. A CentroLB960 plate reader (Berthold) was used for luminescence detection.

### Statistics

All data in bar graphs are means ± SEMs. Data were analyzed using the Student *t* test for comparisons between two groups, and one-way and two-way ANOVA followed by indicated *post hoc* tests for multiple analyses. *P* ≤ 0.05 was considered statistically significant. Analyses were performed using Prism version 10 (GraphPad).

## Supporting information

S1 Fig Memory generalization and altered engram cell activation after spaced training in aged flies. **A)** 24 hours after spaced training, young flies avoid the CS+ odor but not other odors. In contrast, spaced training causes aged flies to avoid multiple odors. *N* = 8–12. *, **, ***, and **** indicate *P* < 0.05, 0.01, 0.001, and 0.0001. **B)** Aging flies at 18 °C for 40 days (the protocol used for obtaining aged flies for memory engram analysis) induces memory generalization similar to flies aged at 25 °C. **C)** Old flies subjected to CS+ training (spaced training using only the CS+ odor and no CS− odor) show a reduced odor avoidance differential between CS+ and novel odors compared to young flies. Generalization is less apparent in this experiment because the novel odor used was already slightly aversive. **D)** Upper panels, the number of GFP+, putative engram cells, produced after paired training, CS+ only training, or odor-only training is similar in young and aged flies. Lower panels, in young flies, exposure to the CS+ odor induces ERK phosphorylation in significantly more GFP+ cells that exposure to a novel odor after paired or CS+ only training. In old flies, exposure to the CS+ or novel odor induces similar pERK activation after paired or CS+ only training. In both young and old flies, odor exposure after control, odor only training (training in the absence of a US), produces similar low numbers of pERK activation in GFP+ cells. The data underlying this figure are available in S1 Data.
(TIF)

S2 Fig Old flies are able to distinguish odors after spaced-training and odor generalization occurs specifically in aged spaced-trained flies. **A)** Prior spaced training does not affect the ability of aged flies to form subsequent short-term memories. (Left side of graph) Memory generalization is observed 24 hours after spaced training in aged flies. (Right side of graph) Aged flies can form normal short-term memories (STM) 24 hours after spaced training, indicating that aged flies are able to distinguish odors after spaced training. *N* = 6. **B)** Memory generalization does not occur after massed training. Twenty-four hours after massed training, both young and aged flies avoided the CS+ odor but not the CS− odor. *N* = 6. **C)** *rsh* mutants, which are defective for anesthesia-resistant memory, show an age-dependent memory generalization after spaced training similar to wild-type flies. *N* = 4–8. The data underlying this figure are available in S1 Data.
(TIF)

S3 Fig *Eaat1* expression increases after spaced training in young flies, and spaced training increases activity of a subset of dopaminergic neurons in aged but not young flies. **A)** *eaat1* expression increases after spaced-training and this increase becomes significant 3 hours after training. **B)** Numbers of tyrosine hydroxylase- and phospho-S6-double positive neurons observed in brains of indicated flies. A significant increase was observed after spaced-training of aged flies. *N* = 7–8. *, **, ***, and **** indicate *P* < 0.05, 0.01, 0.001, and 0.0001. **C)** No significant differences in tyrosine hydroxylase-positive, phospho-S6-positive neurons were observed after massed training. *N* = 4–7. **D)** Glial overexpression of *eaat1* significantly reduced the number of tyrosine hydroxylase-positive, phospho-S6-positive neurons observed after spaced training in aged flies. *N* = 5–7. **E)** Representative images showing increased phospho-S6 in MB060B dopaminergic neurons 24 hours after spaced training in old, but not young flies. The data underlying this figure are available in S1 Data.
(TIF)

S4 Fig Expression of an *NR1* Mg^2^+ block mutant induces generalization, while NR1 knockdown prevents generalization. **A)** Left side, young. Overexpression of an NMDAR Mg^2+^ block mutant in PPL1 neurons (MB060B/NR1 N631Q) induces a spaced training-dependent avoidance to IAA, an odor not associated with training, in young flies. Right side, old. Knockdown of NMDARs prevents training-dependent avoidance of IAA in aged flies. *N* = 5–11. *, ***, and **** indicate *P* < 0.05, 0.001, and 0.0001. **B)** Aging is not associated with a reduction in extracellular Mg^2+^ concentrations. Concentrations of indicated cations were measured in the head hemolymph of young and aged flies as described in Raccuglia and colleagues, 2019. *N* = 6–16. * and ** indicate P < 0.05 and 0.01. The data underlying this figure are available in S1 Data.
(TIF)

S5 Fig Knockdown of *Dop1R2* or *DopEcR* does not affect memory generalization. **A)** Left panel, LTM scores of young and old *tub-GAL80*^*ts*^*/+; kayak-GAL4/UAS-Dop1R2RNAi* flies trained at GAL80^ts^ permissive (−hs) or restrictive (+hs) temperatures. Knocking down *Dop1R2* did not affect LTM in old flies. *N* = 6–14. Right panel, avoidance of the CS+ and CS− odors in these flies. Knocking down *Dop1R2* did not affect memory generalization in old flies. *N* = 5. **B)** Left panel, LTM scores of young and old *tub-GAL80*^*ts*^*/+; kayak-GAL4/UAS-DopEcRRNAi* flies trained at GAL80^ts^ permissive (−hs) or restrictive (+hs) temperatures. Knocking down *DopEcR* did not affect LTM in old flies. *N* = 5–6. Right panel, avoidance of the CS+ and CS− odors in these flies. Knocking down *DopEcR* did not affect memory generalization in old flies. *N* = 5. The data underlying this figure are available in S1 Data.
(TIF)

S6 Fig Odor responses in α2sc-MBON are altered in aged flies. Upper panels, α2sc-MBON Ca^2+^ responses to the CS+ odor are significantly higher than responses to a novel odor, IAA, 24 hours after training in young flies. Ca^2+^ responses to the CS+ and IAA are not significantly different after training in old flies. The gray bar above each graph indicates the duration odor exposure. *N* = 9. Lower left panel, quantification of the log_10_ of the CS+/IAA peak response ratio. Lower right panel, quantification of average peak responses. The data underlying this figure are available in S1 Data.
(TIF)

S7 Fig A model for memory generalization and age-related long-term memory impairments. Our data suggest that there is a cellular pathway linking glutamatergic neurons, PPL1 dopaminergic neurons, and memory engram cells, which is important for regulating the specificity of engram cell reactivation and, consequently, memory specificity. Activity in this pathway needs to be inhibited after spaced training during LTM consolidation to maintain specificity and prevent generalization. In young flies, this occurs because spaced training induces glial expression of the glutamate transporter EAAT1, which reduces glutamate signaling to PPL1 neurons, thereby reducing activation of Dop2Rs on engram cells. Old flies are not able to reduce activity of this pathway because they have reduced amounts of Klg and Repo, which are required for EAAT1 expression. While we are not certain how activation of Dop2Rs affects engram cell plasticity, our data are consistent with a model where reactivation of engram cells during consolidation reinforces memory specificity, while inhibition of engram cell activity during consolidation enhances engram cell connectivity to non-specific inputs.
(TIF)

S1 Data Data summary. Excel file containing the individual data points used for all bar graphs in this manuscript (Figs 1–7 and S1–S6). For line graphs with error bars in Figs 4A, 7A, 7B, 7C, 7D, and S6, numerical values for averages and SEMs for each time point are provided.
(XLSX)

## Abbreviations

AI: avoidance index
AMI: age-related memory impairment
ARM: anesthesia-resistant memory
ATR: all-trans retinal
BEN: benzaldehyde
GS: geneswitch
IAA: Isoamylacetate
Klg: Klingon
Lola: longitudinals lacking
LTM: long-term memory
MCH: 4-methylcyclohexanol
MTM: middle-term memory
PI: performance index
rsh: radish
PTSD: post-traumatic stress disorder
US: unconditioned stimulus
OCT: 3-octanol
3IY: 3-iodotyrosine
