# Supplementary figures and images for: Aberrant dopaminergic activity during consolidation causes age-related memory generalization in Drosophila
Source: PLoS Biol. 2026 Apr 1;24(4):e3003752. doi: 10.1371/journal.pbio.3003752 (PMC13065333; doi:10.1371/journal.pbio.3003752)

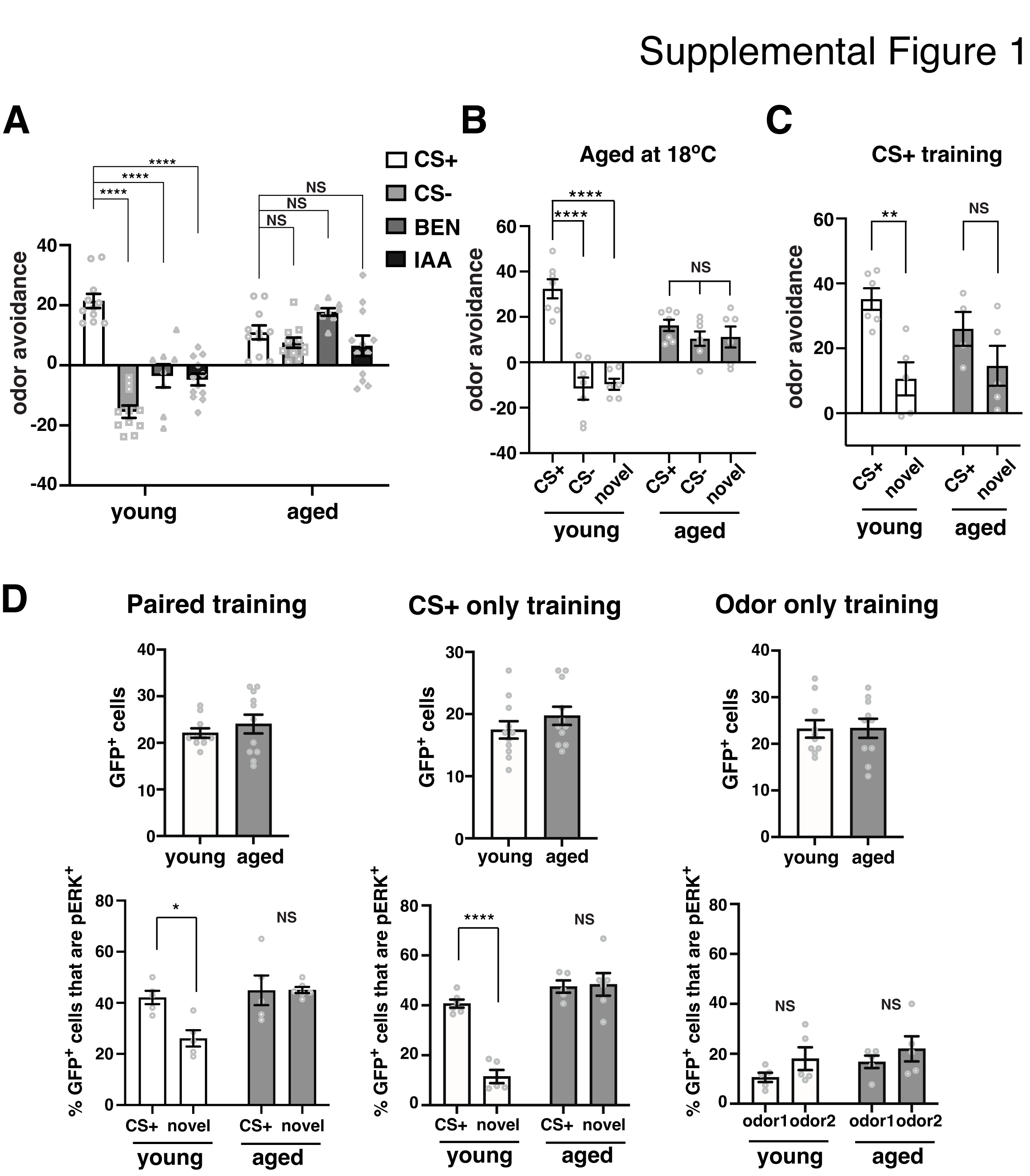

Supplement: S1 Fig — A) 24 hours after spaced training, young flies avoid the CS+ odor but not other odors. In contrast, spaced training causes aged flies to avoid multiple odors. N = 8–12. *, **, ***, and **** indicate P < 0.05, 0.01, 0.001, and 0.0001. B) Aging flies at 18 °C for 40 days (the protocol used for obtaining aged flies for memory engram analysis) induces memory generalization similar to flies aged at 25 °C. C) Old flies subjected to CS+ training (spaced training using only the CS+ odor and no CS− odor) show a reduced odor avoidance differential between CS+ and novel odors compared to young flies. Generalization is less apparent in this experiment because the novel odor used was already slightly aversive. D) Upper panels, the number of GFP+, putative engram cells, produced after paired training, CS+ only training, or odor-only training is similar in young and aged flies. Lower panels, in young flies, exposure to the CS+ odor induces ERK phosphorylation in significantly more GFP+ cells that exposure to a novel odor after paired or CS+ only training. In old flies, exposure to the CS+ or novel odor induces similar pERK activation after paired or CS+ only training. In both young and old flies, odor exposure after control, odor only training (training in the absence of a US), produces similar low numbers of pERK activation in GFP+ cells. The data underlying this figure are available in S1 Data. (TIF) [file pbio.3003752.s001.tif]

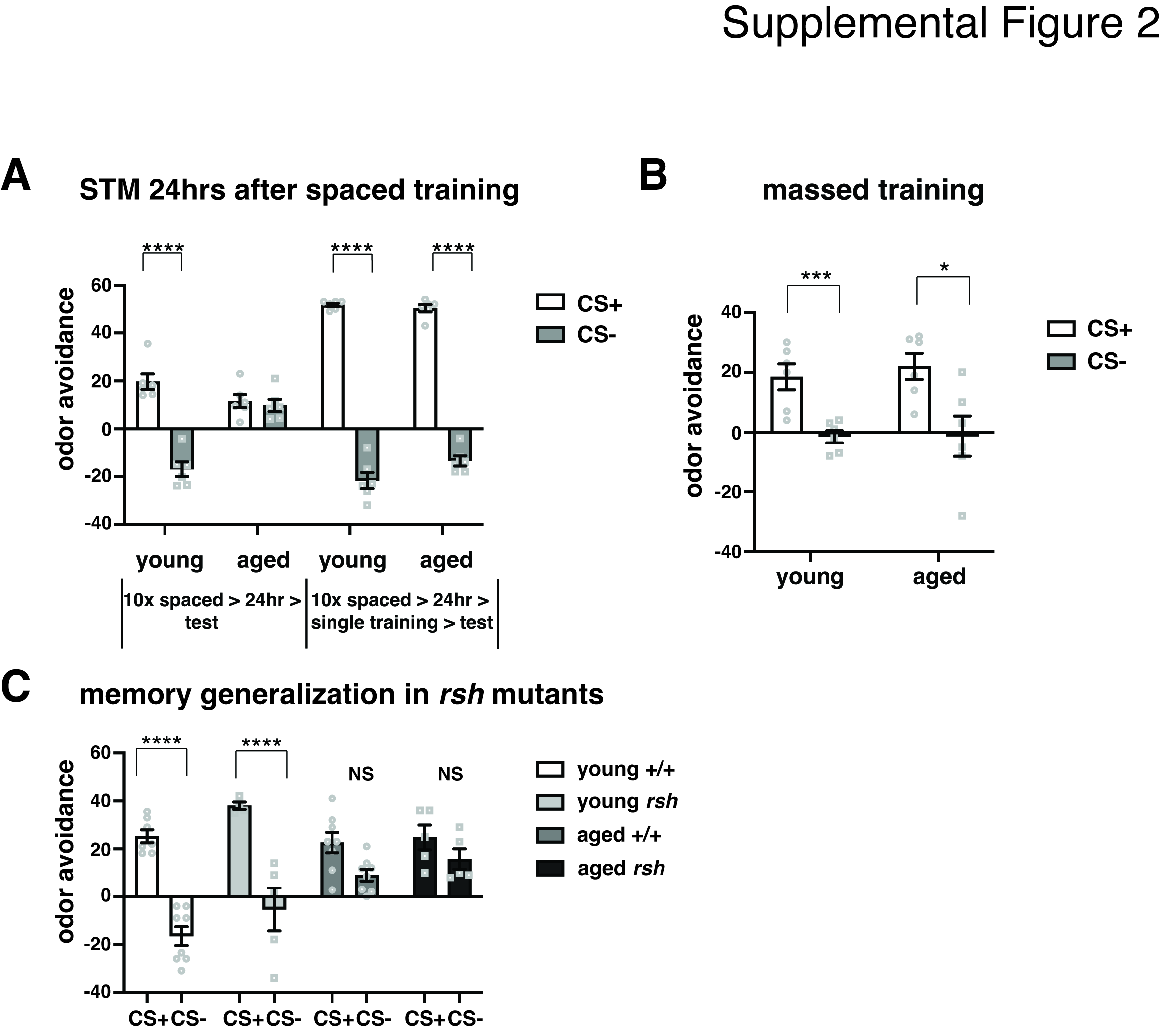

Supplement: S2 Fig — A) Prior spaced training does not affect the ability of aged flies to form subsequent short-term memories. (Left side of graph) Memory generalization is observed 24 hours after spaced training in aged flies. (Right side of graph) Aged flies can form normal short-term memories (STM) 24 hours after spaced training, indicating that aged flies are able to distinguish odors after spaced training. N = 6. B) Memory generalization does not occur after massed training. Twenty-four hours after massed training, both young and aged flies avoided the CS+ odor but not the CS− odor. N = 6. C) rsh mutants, which are defective for anesthesia-resistant memory, show an age-dependent memory generalization after spaced training similar to wild-type flies. N = 4–8. The data underlying this figure are available in S1 Data. (TIF) [file pbio.3003752.s002.tif]

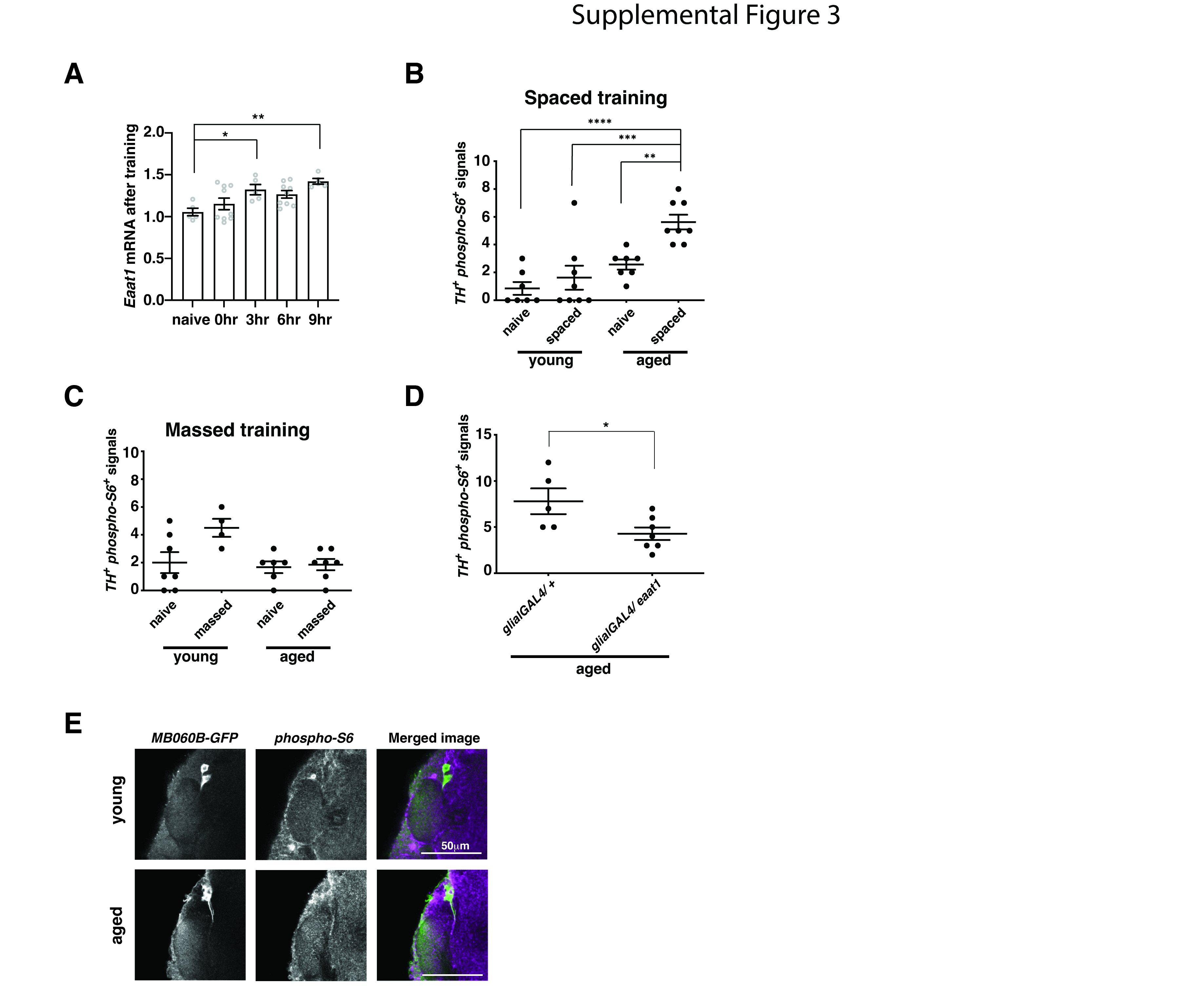

Supplement: S3 Fig — A) eaat1 expression increases after spaced-training and this increase becomes significant 3 hours after training. B) Numbers of tyrosine hydroxylase- and phospho-S6-double positive neurons observed in brains of indicated flies. A significant increase was observed after spaced-training of aged flies. N = 7–8. *, **, ***, and **** indicate P < 0.05, 0.01, 0.001, and 0.0001. C) No significant differences in tyrosine hydroxylase-positive, phospho-S6-positive neurons were observed after massed training. N = 4–7. D) Glial overexpression of eaat1 significantly reduced the number of tyrosine hydroxylase-positive, phospho-S6-positive neurons observed after spaced training in aged flies. N = 5–7. E) Representative images showing increased phospho-S6 in MB060B dopaminergic neurons 24 hours after spaced training in old, but not young flies. The data underlying this figure are available in S1 Data. (TIF) [file pbio.3003752.s003.tif]

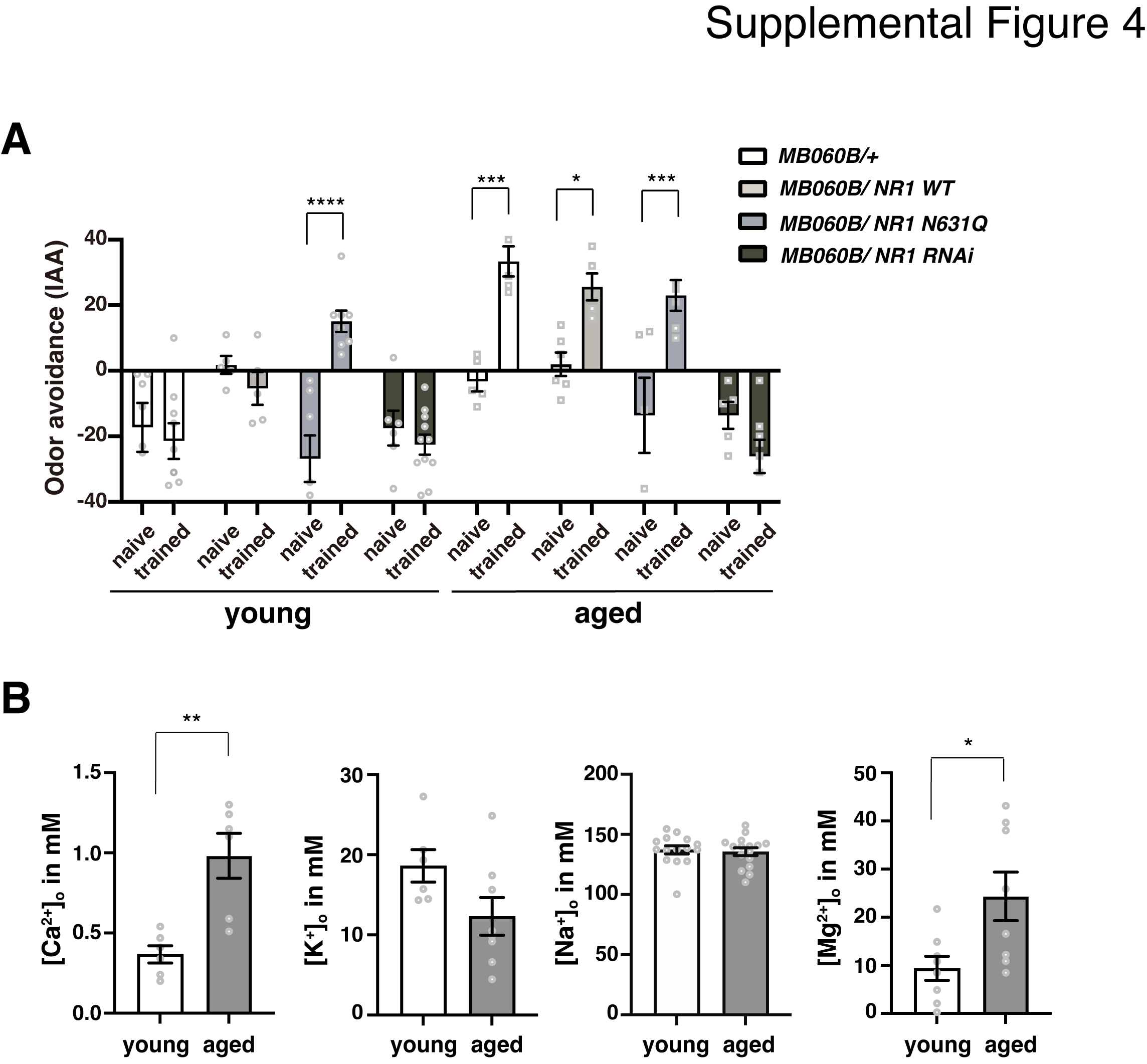

Supplement: S4 Fig — A) Left side, young. Overexpression of an NMDAR Mg2+ block mutant in PPL1 neurons (MB060B/NR1 N631Q) induces a spaced training-dependent avoidance to IAA, an odor not associated with training, in young flies. Right side, old. Knockdown of NMDARs prevents training-dependent avoidance of IAA in aged flies. N = 5–11. *, ***, and **** indicate P < 0.05, 0.001, and 0.0001. B) Aging is not associated with a reduction in extracellular Mg2+ concentrations. Concentrations of indicated cations were measured in the head hemolymph of young and aged flies as described in Raccuglia and colleagues, 2019. N = 6–16. * and ** indicate P < 0.05 and 0.01. The data underlying this figure are available in S1 Data. (TIF) [file pbio.3003752.s004.tif]

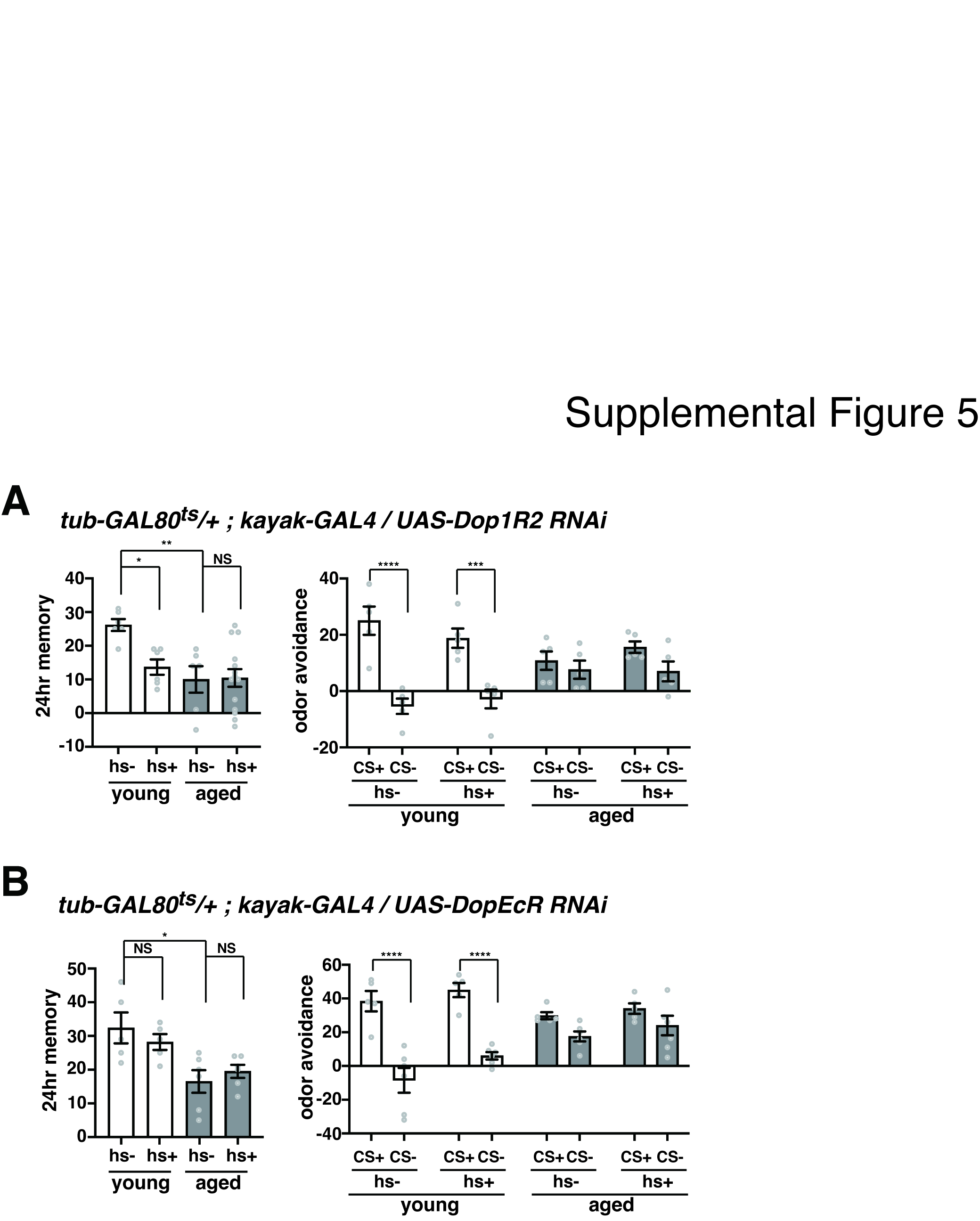

Supplement: S5 Fig — A) Left panel, LTM scores of young and old tub-GAL80ts/+; kayak-GAL4/UAS-Dop1R2RNAi flies trained at GAL80ts permissive (−hs) or restrictive (+hs) temperatures. Knocking down Dop1R2 did not affect LTM in old flies. N = 6–14. Right panel, avoidance of the CS+ and CS− odors in these flies. Knocking down Dop1R2 did not affect memory generalization in old flies. N = 5. B) Left panel, LTM scores of young and old tub-GAL80ts/+; kayak-GAL4/UAS-DopEcRRNAi flies trained at GAL80ts permissive (−hs) or restrictive (+hs) temperatures. Knocking down DopEcR did not affect LTM in old flies. N = 5–6. Right panel, avoidance of the CS+ and CS− odors in these flies. Knocking down DopEcR did not affect memory generalization in old flies. N = 5. The data underlying this figure are available in S1 Data. (TIF) [file pbio.3003752.s005.tif]

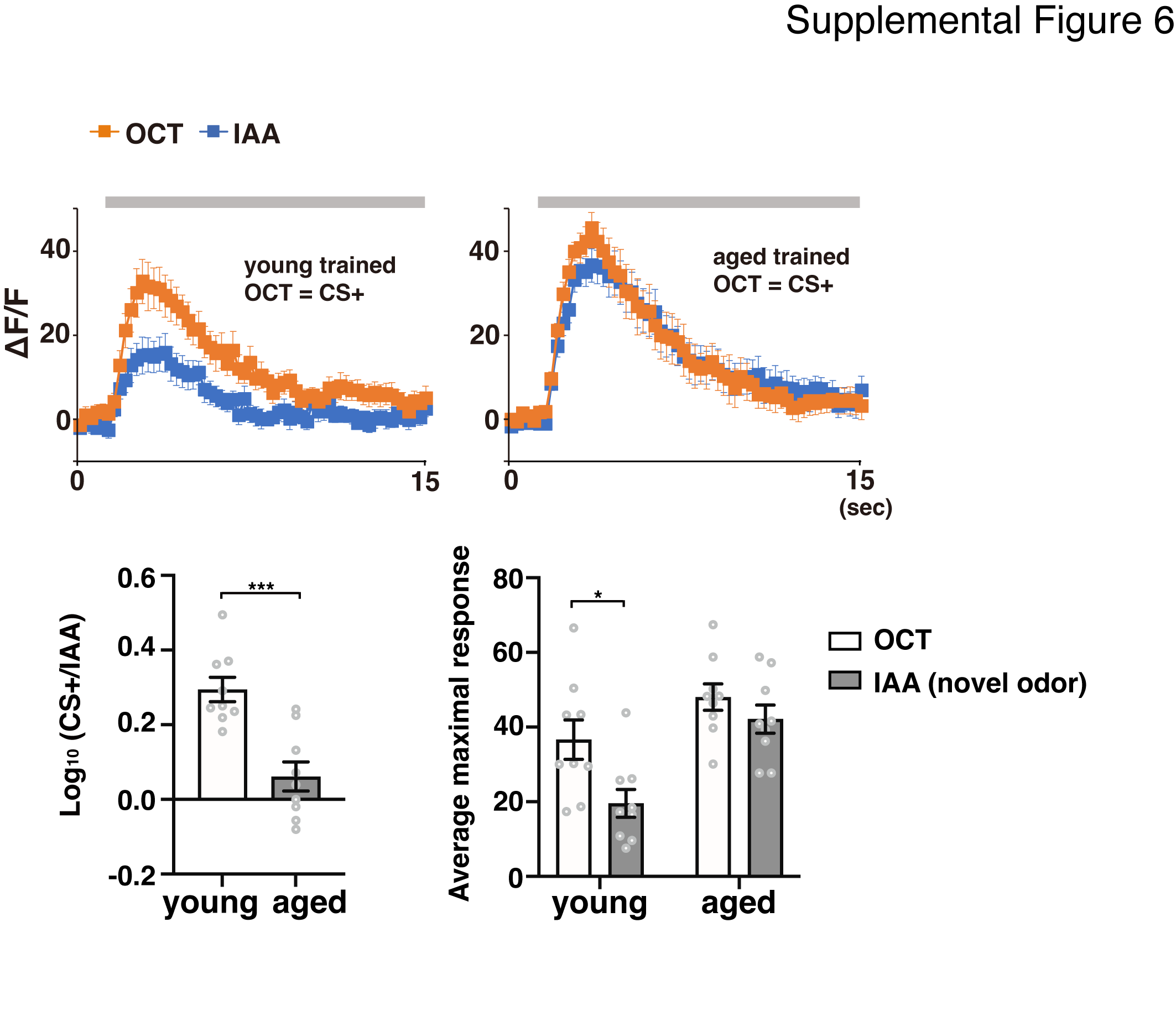

Supplement: S6 Fig — Upper panels, α2sc-MBON Ca2+ responses to the CS+ odor are significantly higher than responses to a novel odor, IAA, 24 hours after training in young flies. Ca2+ responses to the CS+ and IAA are not significantly different after training in old flies. The gray bar above each graph indicates the duration odor exposure. N = 9. Lower left panel, quantification of the log10 of the CS+/IAA peak response ratio. Lower right panel, quantification of average peak responses. The data underlying this figure are available in S1 Data. (TIF) [file pbio.3003752.s006.tif]

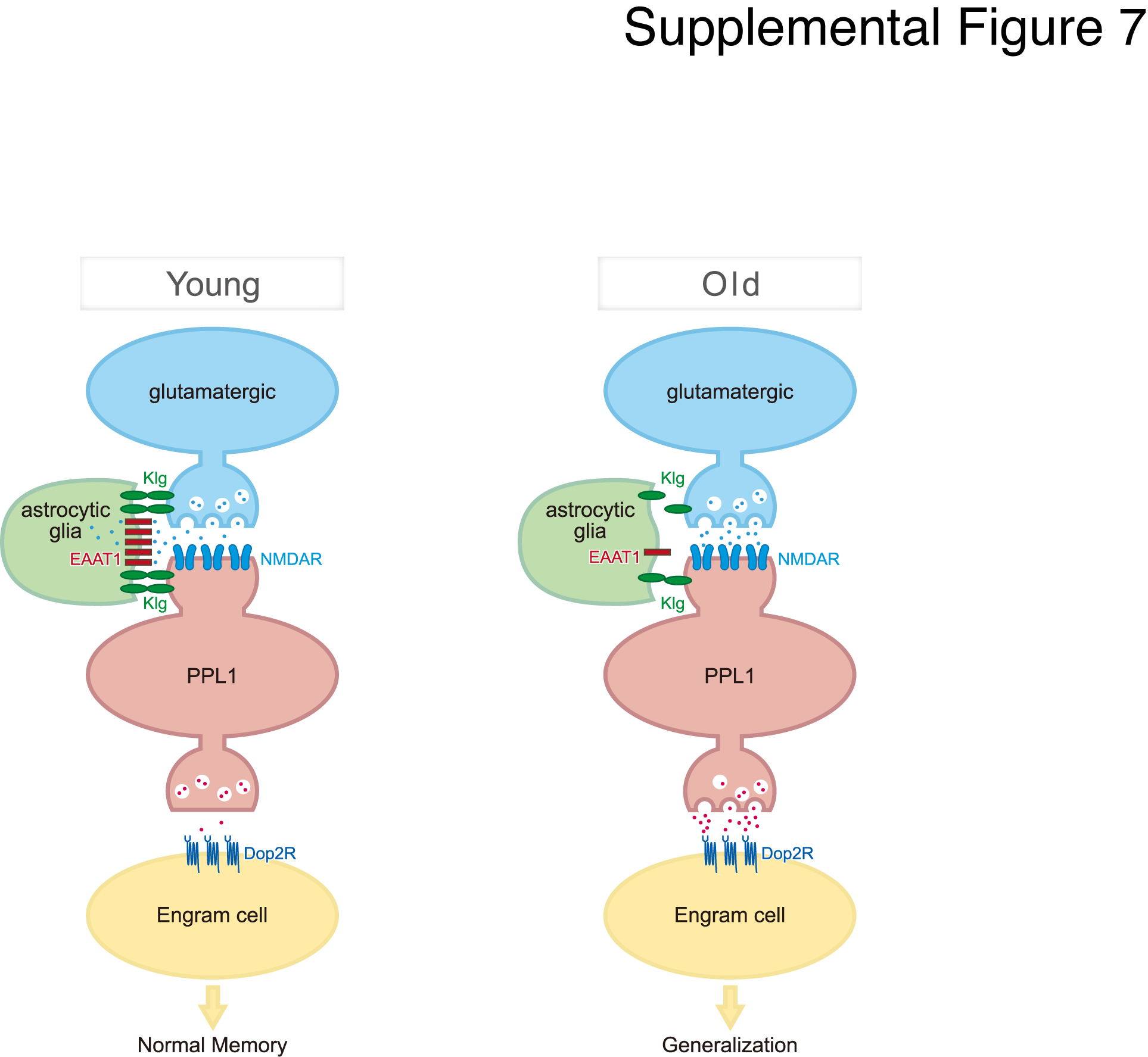

Supplement: S7 Fig — Our data suggest that there is a cellular pathway linking glutamatergic neurons, PPL1 dopaminergic neurons, and memory engram cells, which is important for regulating the specificity of engram cell reactivation and, consequently, memory specificity. Activity in this pathway needs to be inhibited after spaced training during LTM consolidation to maintain specificity and prevent generalization. In young flies, this occurs because spaced training induces glial expression of the glutamate transporter EAAT1, which reduces glutamate signaling to PPL1 neurons, thereby reducing activation of Dop2Rs on engram cells. Old flies are not able to reduce activity of this pathway because they have reduced amounts of Klg and Repo, which are required for EAAT1 expression. While we are not certain how activation of Dop2Rs affects engram cell plasticity, our data are consistent with a model where reactivation of engram cells during consolidation reinforces memory specificity, while inhibition of engram cell activity during consolidation enhances engram cell connectivity to non-specific inputs. (TIF) [file pbio.3003752.s007.tif]
